# Evaluating the relationship between the nutrient intake of lactating women and their breast milk nutritional profile: a systematic review and narrative synthesis

**DOI:** 10.1017/S0007114523002775

**Published:** 2024-04-14

**Authors:** Coralie Falize, M. Savage, Yvonne M. Jeanes, Simon C. Dyall

**Affiliations:** School of Life and Health Sciences, University of Roehampton, London, UK

**Keywords:** Lactating mothers, Human milk, Milk banks, *n*-3 PUFA, Nutrients, Vegan, Vegetarian

## Abstract

Maternal diet influences breast milk nutritional profile; however, it is unclear which nutrients and contaminants are particularly responsive to short- and long-term changes in maternal intake, and the impact of specific exclusion diets, such as vegan or vegetarian. This study systematically reviewed the literature on the effects of maternal nutrient intake, including exclusion diets, on both the nutrient and contaminant content of breast milk. The electronic databases, PubMed, CENTRAL, Web of Science and CINALH were systematically searched until 4 June 2023, with additionally searches of reference lists (PROSPERO, CRD42020221577). The quality of the studies was examined using Cochrane Risk of Bias tool and Newcastle–Ottawa scale. Eighty-eight studies (*n* 6577) met the search criteria. Due to high heterogeneity, meta-analysis was not possible. There was strong evidence of response to maternal intakes for DHA and EPA, vitamins A, E and K, iodine and Se in breast milk composition, some evidence of response for *α*-linolenic acid, B vitamins, vitamin C and D, ovalbumin, tyrosine and contaminants, and insufficient evidence to identify the effects arachidonic acid, Cu, Fe, Zn and choline. The paucity of evidence and high heterogeneity among studies reflects the need for more high-quality trials. However, this review identified the importance of maternal intake in the nutritional content of breast milk for a wide range of nutrients and supports the recommendation for supplementation of DHA and vitamin B_12_ for those on restrictive diets.

Appropriate nutrition is fundamental for newborns, especially preterm infants, where their materno-fetal nutrients supply have been prematurely terminated^(1)^. Breast milk provides nutrients, hormones, enzymes and immunological factors that are essential for infant development. Maternal breast milk is the first choice for feeding neonates; however, if mothers are unable to provide sufficient, donor human milk is the recommended alternative^(2)^. The nutritional composition of human milk varies widely, not only over lactation, but also between individuals and populations^(3–5)^. Factors that have been shown to affect the nutritional composition include maternal lifestyle and dietary habits^(6)^. It is therefore essential to identify which nutrients in breast milk are responsive to maternal diet in order to inform and update nutritional guidance for lactating mothers, milk donors and milk banks.

The impact of maternal diet on breast milk composition has been widely investigated; however, the results have been equivocal, with some studies showing positive effects whereas others have not. Systematic reviews in 2016^(6)^ and 2017^(7)^ report a positive association between oily fish consumption and higher levels of the *n*-3 PUFA, DHA (22:6*n*-3) and EPA (20:5*n*-3), and other fatty acids, such as the *n*-6 PUFA, linoleic acid (LA, 18:3*n*-6), and oleic acid (18:1*n*-9) in breast milk^(6)^. There was also evidence demonstrating a positive association between dietary vitamin C, B_1_ and vitamin A, D, E and K, with breast milk levels. The effects of dietary vitamin and/or mineral supplementation were reported in two systematic reviews^(8,9)^, where the results were mixed, although there was some indication that vitamin supplementation had a greater effect on breast milk levels than mineral supplementation, with the strongest evidence seen for vitamin A, D, B_1_, B_2_, B_12_ and C.

Restricted diets, such vegan or vegetarian, can provide lower levels of nutrients, such as DHA, which may consequently affect the nutritional content of breast milk. In a 2020 systematic review by Karzc and Krόlak-Olejnik, the effects of vegan or vegetarian diets on breast milk composition were explored^(8)^. Thirteen studies were summarised, and the authors identified that although milk from mothers following vegan, vegetarian and non-vegetarian diets was generally comparable in nutritional content, there were lower levels of some nutrients, particularly of DHA and vitamin B_12_, in the milk from vegan mothers and, therefore, recommended supplementation with these nutrients to enhance the nutritional content of the milk.

DHA, the long-chain *n*-6 PUFA and arachidonic acid (ARA, 20:4*n*-6) are essential for the development of optimal brain, visual and immune system functions^(10)^. In addition to those following vegan diets, lactating women may limit their consumption of fish, where DHA and EPA are highly enriched, due to concerns over the presence of heavy metal contaminants, such as methylmercury^(11)^ and their effects on breast milk composition. This review also investigated the effects of dietary exposure to contaminants including heavy metals (As, B, Pb and Hg) and polychlorinated biphenyls (PCB) on breast milk levels.

The aim of this review is to extend the scope of previous systematic reviews and provide an up-to-date summary of the effects of short-term and long-term changes in maternal nutrient intake, including restrictive diets, and on breast milk nutritional composition. It is hoped that the results may be used to guide future research and inform nutritional guidance for lactating mothers, milk donors and milk banks.

## Methodology

This review was designed and undertaken following the protocols for Preferred Reporting Items for Systematic Review and Meta-Analysis (PRISMA)^(12)^. Study selection, assessment of eligibility, extraction of data and statistical analysis were performed according to a predefined protocol registered with the PROSPERO International prospective register of systematic reviews (ID: CRD42020221577).

### Search strategy

The search was performed on four different databases: PubMed, CENTRAL, Web of Science and CINALH following the PRISMA^(12)^ statement for systematic reviews. Additionally, three systematic reviews^(6,8,9)^ were screened and articles meeting the selection criteria were also included. The review was designed upon the participants/population, intervention, comparison and outcome (PICO) model, with population being ‘lactating mothers’ or ‘human milk donors’; intervention, ‘experimental’ or ‘observation’ studies; comparison, ‘maternal dietary intake’ and outcome, ‘micronutrients’, ‘macronutrients’ and ‘contaminants’ breast milk content. The search was conducted on human studies and exclusively on lactating women. Publication types included were randomised controlled trials (RCT), experimental studies and observational studies. Limitations were applied to exclude conference papers, editorials, letters, commentary, and short survey, and grey literature was not searched. The search was run in English language up to 4 June 2023, with no time limitation. Online Supplementary Table S1 shows the search strategy.

### Selection criteria

The selection criteria were based on the participants/population, intervention, comparison and outcome framework^(13)^. The participants/population were healthy, non-micronutrient-deficient, lactating women, as defined by investigators. The participants/population were breast-feeding or expressing breast milk within the first 12 months postpartum, and the exclusion criteria were participants/populations with predisposition to malnutrition, micronutrient deficiency as defined by investigators, any severe medical conditions or disorders including, HIV 1 or 2, hepatitis B or C, human T-lymphotropic virus type I or II, or syphilis, recreational drug users, smokers, or users of nicotine replacement therapy. The intervention was dietary supplementation for RCT and experimental studies, and assessment of dietary intake for observational studies. The comparison was to the control group or differences in relative levels of intake. The outcome was the differences in breast milk nutrient/contaminant level by maternal intake.

### Data extraction

Two reviewers independently screened all titles and abstracts (CF and MS), according to the inclusion and exclusion criteria. Disagreements were resolved by discussion and where necessary involving a third reviewer (SCD). If the title or abstract appeared to meet the eligibility criteria or they could not determine its eligibility, the full texts of the articles were obtained. Full-text screenings and quality assessments for each of the included papers were also conducted by the two reviewers independently (CF and MS), and any discrepancies were discussed with a third author (SCD) until a decision on whether or not to include the paper in the review was reached. Rayyan software was used for handling and managing extracted studies that were found in the databases, and duplicates were removed^(14)^.

### Quality assessment and risk of bias

The quality assessment of the studies was performed by using the Cochrane Risk of Bias tool (ROB2_IRPG_beta_v7)^(15,16)^ for RCT and the Newcastle–Ottawa scale for the non-randomised cohort and case–control studies^(17)^. The ROB2_IRPG_beta_v7 assessment tool contains five domains: randomisation process, deviations from intended interventions, missing outcome data, measurement of the outcome and selection of the reported result. An algorithm calculates the risk of bias for each domain as well as the overall risk, classifying it within three categories, high risk, low risk or some concerns. The Newcastle–Ottawa scale is comprised of eight items covering three domains: selection (including representativeness and source of sample), comparability (including study design and considerations in analysis) and exposure (for cohort studies, the exposure domain is instead the ‘outcome’ domain). Each paper can be assigned a score of 9 stars and was rated as either ‘good’, ‘fair’ or ‘poor’. The quality of each study was rated using the following scoring algorithms: ≥ 7 points were considered as ‘good’, 3–6 points were considered as ‘fair’ and ≤ 2 points was considered as ‘poor’ quality.

### Statistical analysis

Information was extracted on author, type of study, geographical area, characteristics and number of participants, evaluated nutrients, type of supplement when intervention, breast milk extraction method, aim and outcome of the study, and most relevant findings. Among the examined nutrients in breast milk, results are reported for heavy metals, iodine, Fe, Cu, *n*-3 and *n*-6 PUFA, ovalbumin, persistent organic pollutants, protein, retinol, Se, vitamin A, B vitamins, vitamin B, vitamin C, vitamin D, vitamin E, vitamin K and Zn. A random effect meta-analysis was conducted with RevMan 5.4, Cochrane’s online review-writing platform on fatty acids (DHA, EPA and ARA), vitamins A, D and E, iodine and Se; however, due to high heterogeneity, it was decided not to publish the meta-analysis results. In the final summary, the overall certainty of the evidence was rated by the authors as either (1) very low, (2) low, (3) moderate or (4) high, following the Grading of Recommendations Assessment, Development and Evaluation system^(18)^.

## Results

### Description of the identified studies

**Fig. 1.**
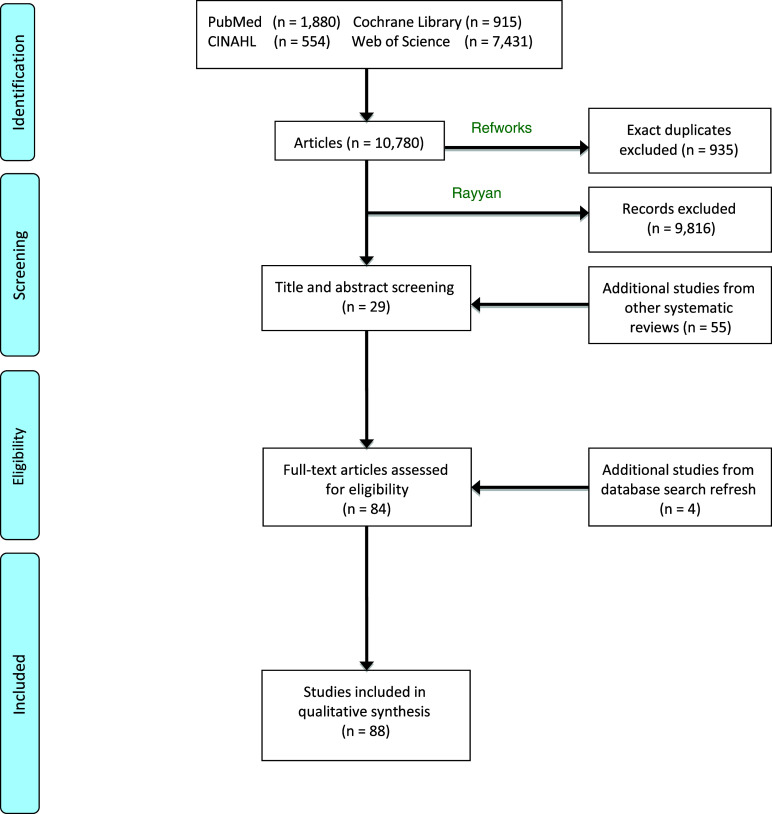
PRISMA 2009 flow diagram. From: Moher D, Liberati A, Tetzlaff J, Altman DG, The PRISMA Group (2009). Preferred Reporting Items for Systematic Reviews and Meta-Analyses: The PRISMA Statement. PLoS Med 6(7): e1000097. doi:10.1371/journal.pmed1000097. For more information, visit www.prisma-statement.org.

The initial search identified 10 780 articles across four databases: PUBMED, CENTRAL, CINAHL and Web of Science; Fig. 1 shows the article selection procedure (PRISMA flow chart): 10 702 articles were excluded, 935 because they were duplicates, and 9816 after abstract and title screening. Fifty-nine additional articles were identified from reference lists.

In total, eighty-eight articles were included in the final review, fifty-four experimental studies and thirty-four observational, comprising a total of 6577 participants. Twenty-nine articles examined fatty acids, thirty-one examined vitamins, twenty-three examined minerals, amino acids and proteins, and five examined contaminants (heavy metals: As, B, Pb and Hg) and PCB. For the rest of the nutrients, there were less than five studies each. The breakdown per nutrient is shown in online Supplementary Table S2.

### Breast milk extraction

The breast milk collection details are summarised in Tables 1–4. The other studies either analysed samples taken over the day or did not specify the time of collection.

**Table 1.** Responsivity of breast milk fatty acid content to maternal diet

Ref	Participants Risk of bias	Study	Ethnicity	Breast milk collection timing	Effects on breast milk PUFA content
Experimental studies					
Argaw, 2020	Healthy lactating women 6–12 months PP Ethiopia RCT *Some concerns*	215 mg DHA and 285 mg EPA per d (*n* 72) or placebo (control maize oil without DHA or EPA, *n* 82) for 6 months	African	Not provided	Supplementation increased BM DHA, by 39 % (95 % CI 20·6, 57·5 %, *P* < 0·001) and EPA by 36 % (95 % CI 16·0, 56·4 % *P* < 0·001) compared with controls
Boris, 2004	Healthy lactating women enrolled at 30 weeks GA Denmark RCT *Some concerns*	Fish oil supplementation (900 mg DHA and 300 mg EPA per d) until delivery (*n* 12), or further 30 d (*n* 11), or placebo (olive oil, *n* 13)	Danish women supposedly White Caucasian	Morning milk from one breast only	BM DHA levels 2·1, 3·6 and 2·8 times higher at 4, 16 and 30 d, respectively, in extended supplementation group *v*. control group (all *P* < 0·001)
Craig-Schmidt, 1984	Healthy lactating women 2 months PP USA RCT *Some concerns*	BM samples (*n* 8). Participants provided with a two 5 d diets with an intervening 2 d period. Diets for the two periods were identical except that source of hydrogenated fats were used in the first period and non-hydrogenated fats is the second period	Not provided	Milk samples collected after first nursing of the day by manual expression for both breasts	BM myristic acid (14:0), palmitic acid (16:0) and palmitoleic acid (16:1*n*-7), all significantly lower following diets with hydrogenated *v*. non-hydrogenated fats, whereas elaidic acid (18:1 trans-9) and oleic acid (18:1*n*-9) were significantly higher
Fougere, 2021	Healthy lactating women, < 72 h PP France RCT Low risk of bias	1·2 g DHA per d (*n* 196) or placebo (maize oil and soyabean oil, (*n* 193) for 14 d	Not provided	Not provided	After 14 d, significantly higher levels of BM mean (sd) DHA and EPA in supplemented (DHA: 0·95 % (0·44 %); EPA: 0·08 %, (0·05 %)) *v*. control (DHA: 0·34 %, (0·20 %); EPA: 0·07 %, (0·07 %)), both *P* < 0·0001
Hawkes, 2002	Healthy lactating women 3 d PP Australia RCT *Some concerns*	300 mg DHA and 70 mg EPA per d (*n* 26), or 600 mg DHA and 140 mg EPA per d (*n* 28), or placebo (sunflower oil, *n* 27) for 4 weeks	Not provided	Hand-expressed morning milk	Mean (sd) BM DHA content increased in a linear manner in response to dietary DHA (placebo DHA: 0·26 %, (0·08 %); LoDHA: 0·39 %, (0·09 %); HiDHA: 0·66 %, (0·18 %); all *P* < 0·05). BM EPA only significantly increased in HiDHA group (placebo EPA: 0·11 %, (0·02 %); LoDHA: 0·11 %, (0·02 %); HiDHA: 0·14 %, (0·03 %); *P* < 0·05). No significant effects on ARA
Lauritzen2002	Healthy lactating women 4 months PP Denmark RCT *Some concerns*	BM samples (*n* 12). Lactating women were given fish oil (2–8 g) for breakfast and delivered 6–12 BM samples during the following 24 h	Not provided	Morning milk, after first feeding	Mean (sd) BM DHA of the fish-eating mothers was 0·57 %, (0·28 %) and non-fish-eating mothers was 0·42 %, (0·15 %); *P* = 0·05. Fish oil supplementation resulted in a 2-fold increase in BM DHA levels, peaked after 10 h and lasted for 24 h
Mazurier, 2017	Lactating mothers 1–4 months PP France RCT *Low risk of bias*	All groups received 350 mg DHA and 210 mg EPA per d but varied in ALA and LA content. *n* 19–22 per group, with 15 d washout period followed by 15 d supplementation	White Caucasian	Human milk collected at the first infant feeding of the morning	Significant dose response following in ALA (*P* < 0·003), but no significant effects on LA. No significant differences in DHA or EPA between groups. ARA content significantly increased only in intermediate ALA and LA dose, and *n*-3 PUFA-enriched rapeseed oil group
Mellies, 1979	Overweight and normal weight lactating women 1 month PP USA RCT *Some concerns*	BM samples (*n* 14), 2 weeks baseline nutrition history collected by a nutritionist, mothers randomly assigned to one diet followed by the other; diet 1: cholesterol-poor phytosterol-rich, PUFA-rich (PUFA:SFA ratio 1·8); diet 2: cholesterol-rich, phytosterol-poor, PUFA-poor (PUFA:SFA ratio 0·12)	Not provided	Samples collected at the beginning or end of the second nursing period of the day, through manual expression or breast pump	Mean (s em) for baseline diet *v*. diet 1 *v*. diet 2; milk cholesterol (mg/g milk fat) 2·4 (0·4) *v*. 2·4 (0·1) *v*. 2·5(0·2): milk phytosterols (mg/g milk fat) 0·17 (0·03) *v*. 2·2 (0·3) *v*. 0·7 (0·1); total milk fat, 3·58 (0·56) *v*. 2·69 (0·16) *v*. 2·66 (0·16) (*P* < 0·001)
Nasser, 2010	Healthy lactating women between 2 and 6 months PP, vegetarian excluded Canada RCT *Some concerns*	BM samples (*n* 14), low-fat diet or high-fat diet for 4 d in randomised order	Not provided	Milk collected on the last 2 d of each 4-d period between 1 and 14.00 using a manual breast pump	Significant differences in mean (sem) BM composition in low fat *v*. high-fat diet for lauric acid (12:0) 5·38 (1·16) *v*. 3·98 (0·37) (*P* = 0·01), palmitoleic acid (16:1*n*-7) 1·95 (0·29) *v*. 1·31 (0·23) (*P* = 0·046), ALA 1·22 (0·04) *v*. 0·69 (0·06) (*P* = 0·01), ARA 0·34 (0·01) *v*. 0·30 (0·02) (*P* = 0·02)
Park, 1999	Healthy lactating women between 1 and 26 months PP USA RCT *Some concerns*	Three-week crossover study. Week 1, minimal rumenic acid (18:2 cis-9, trans-11) foods (depletion), then either high-fat dairy food or low-fat dairy food intake for 1 week, then crossover (*n* 8 per group). BM samples (*n* 16), and dietary records during last 3 d of each period and FFQ	Not provided	Not provided	Significantly higher BM (mean (sem), μmol/g lipid) in high-fat dairy *v*. low-fat dairy groups for: rumenic acid (13·5 (1·1) *v*. 8·2 (0·4)), myristic acid (264·7 (34·2) *v*. 195·2 (11·0)), palmitic acid (707·0 (51·5) *v*. (511·3 (16·4)), stearic acid (1055·0 (103·4) *v*. 874·3 (33·3)), oleic acid (1055·0 (103·4) *v*. (874·3 (33·3)) and significantly lower ALA (10·7 (3·6) *v*. 17·6 (1·1)), all *P* < 0·05)
Smithers, 2010	Mothers of preterm infants born 33 weeks GA Australia RCT *Low risk of bias*	900 mg DHA, 195 mg EPA, and 54 mg ARA per d, (*n* 69) or placebo (soyabean oil, *n* 74) for 2 weeks	98 % Caucasian	Not provided	DHA significantly higher in supplemented (M = 1·0 % sd = 0·4 %) *v*. placebo (M = 0·3 % sd = 0·1 %) groups, (*P* < 0·05). No significant differences in EPA or ARA between groups
Storck lindholm, 2012	Obese and normal-weight lactating women after delivery Sweden Obs/RCT *Some concerns*	Control group (BMI < 25 kg/m^2^, *n* 26), Group O (BMI > 30 kg/m^2^, *n* 25) and Group I (BMI > 30 kg/m^2^, *n* 25) were given dietary advice (e.g. eat fish 2–3 times a week) and increase physical activity. BM measured 3 and 10 d, 1 and 2 months	White Caucasian	Not provided	Group O had low fish intake and at baseline had the lowest BM ALA, EPA and DHA (all *P* < 0·01), which was continued across subsequent samples. The ARA: EPA + DHA ratio was significantly higher in Group O across repeated samples (*P* < 0·01), compared with the other groups. Group I levels approached those seen in control group
Valentine, 2013	Milk donors mean lactational age 19 weeks USA RCT *Some concerns*	1 g DHA/d (*n* 69), or placebo (soyabean oil, *n* 74) for 14 d	Not provided	Not provided	Supplementation significantly increased DHA content when expressed as mol wt%, but not in absolute amounts. No significant effect of supplementation on EPA or ARA content
Valenzuela, 2015	Lactating women at delivery and 6 months PP Chile RCT *Some concerns*	10·1 g ALA per d (chia oil, *n* 19) or untreated control group (*n* 21) for 9 months	Hispanic	Not provided	Significant increase in ALA and significant decrease in LA following supplementation *v*. control at all time points (all *P* < 0·05). DHA significantly increased in first 3 months (*P* < 0·05), then no effect, no change in EPA or ARA *v*. control at any time point.
Yang, 2022	Healthy lactating women between 30 and 50 d PP China RCT Some concerns	200 mg DHA per d (*n* 77) or placebo (*n* 60) for 8 weeks	Asian	Breast milk samples collected between 07.30 and 09.00	Absolute GLA (18:3*n*-6), ARA and DHA significantly decreased over the study in control group (*P* < 0·001, *P* = 0·001 and *P* < 0·037, respectively), whereas GLA and DHA were maintained in supplemented group, although there was a significant decrease in ARA (*P* = 0·03). DHA content was significantly higher in supplemented *v*. control group at the end (*P* = 0·012. Similar trends were found when expressed as relative content
Observational studies					
Aitchison, 1977	Healthy lactating women between 4 and 6 months PP USA Obs *Fair Quality 6*	BM samples (*n* 11), recorded food intake for 1 week and saved duplicated food portions consumed on 3 d	Not provided	Experiment 1: five subjects took morning and evening milk samples. Experiment 2: six additional subjects collected morning milk only	Correlation coefficient (P) between PUFA to SFA ratio in maternal diet and milk (% of total FA): 0·46 considering diet and milk in the same evening; 0·43 (*P* < 0·05) considering milk in the next morning In nine of eleven subjects, fluctuation of percent total trans acids in the milk appeared to follow dietary trans changes after a 12–36-h lag period
Antonakou, 2013	Healthy lactating women 1 month PP Greece Obs *Fair Quality 6*	BM samples 1-month PP (*n* 64), 3-month PP (*n* 39), 6-month PP (*n* 24). Three-day dietary record at 1st, 3rd and 6th month PP	Caucasian	Morning milk collected after at least 2 h after previous breast-feeding	BM fat ranged from 26·3 and 30·2 g/l (*P* < 0·05). Strong positive effect found during first month lactation between mother’s PUFA intakes and BM PUFA concentration, *r *= 0·25, *P* < 0·05; *n*-3 fatty acids, *r *= 0·26, *P* < 0·05; DHA *r *= 0·27, *P* < 0·05 and LA, *r *= 0·26, *P* < 0·05, while MUFA intake was strongly correlated with concentration of PUFA, *r *= 0·29, *P* < 0·05; *n*-6, *r *= 0·27, *P* < 0·05 and LA,, *r *= 0·25, *P* < 0·05
Bzikowska, 2019	Healthy lactating women 1 month PP Poland Obs *Fair quality 6*	BM samples (*n* 32) and dietary information 3-d dietary record and FFQ	Not provided	Foremilk and hindmilk collected from four time periods: 06.00–12.00, 12.00–18.00, 18.00–00.00 and 00.00–06.00	Significant positive correlation between fatty fish consumption and DHA (τ_b_ = 0·25, *P* = 0·049), EPA (τ_b_ = 0·27, *P* = 0·03) and ALA (τ_b_ = 0·28, *P* = 0·02). ALA positively correlated with intakes of linseed oil (τ_b_ = 0·3, *P* = 0·01), coconut oil (τ_b_ = 0·29, *P* = 0·02) and milk (τ_b_ = 0·26, *P* = 0·04). EPA positively correlated with pork consumption (τ_b_ 0·29, *P* = 0·02)
Daud, 2013	Healthy lactating women between 15 d and 6 months PP Malaysia Obs *Fair Quality 6*	BM samples (*n* 101). Participants provided a 1-year period FFQ. Sub-experiment, BM samples (*n* 18). Participants provided a 3-d FFQ	Asian	Not provided	The most abundant BM *trans*-fatty acid was linoelaidic acid (mean = 1·44%, sem = 0·60 % fatty acids), which was also the most consumed (mean = 0·07 sem = 0·01, g/100 g of food). Ten food items had an effect on the total BM *trans*-fatty acids (buns, chicken burgers, cheeseburgers, shortening, powdered milk, sweetened milk blended oil mayonnaise, maize oil and ice cream). No association between consumption and BM *trans*-fatty acid levels
De la Presa-Owens, 1996	Healthy lactating women < 1 PP Spain Obs *Poor Quality 2*	BM samples (*n* 40), dietary questionnaire	Not provided	Not provided	Lower BM LA observed between mothers consuming olive oil (*n* 15) or sunflower (*n* 6) as the preferred source of fat (*P* < 0·001). Significant differences in BM DHA and EPA between mothers reporting high, low or no fish consumption (*P* < 0·001)
Freitas, 2019	Healthy lactating women < 3 PP Brazil Obs *Fair quality 2*	Diet quality assessed through a semi-structured questionnaire (*n* 106)	Most participants black/multiracial (82 %)	Sample collected after the first breast-feeding of the morning	Long-chain *n*-3 and *n*-6 PUFA not analysed separately. Total fruits and whole fruits, *r *= −0·302, *r *= 0·283, respectively, both *P* < 0·05
Juber, 2017	Healthy new mothers > 1 week PP USA Obs *Fair quality 7*	BM at baseline (*n* 84), subject received analysis of BM DHA and dietary recommendations (*n* 60) had second sample at 1 month	99 % White Caucasian	Not provided	Those reporting taking DHA supplements (*n* 43) had higher levels than those who did not (0·23 % *v*. 0·15 %, *P* < 0·0001). In second sample, median breast milk DHA content increased from 0·19 % to 0·22 % (*P* < 0·01)
Liu, 2015	Healthy lactating women 22–25 d PP China Obs *Good quality 8*	Dietary intake of lactating women assessed with 24-h dietary recall questionnaire (*n* 514)	Asian	Morning milk, manual expression between 09.00 and 11.00	Significant negative correlation between dietary ALA and BM GLA (18:3*n*-6, r^2^ = −0·201, *P* = 0·03) and adrenic acid (22:4*n*-6, r^2^ = −0·197, *P* = 0·03), and dietary LA and BM DGLA (20:3*n*-6, r^2^ = −0·182, *P* = 0·03)
Makela, 2013	Overweight and normal-weight lactating women 3 months PP Finland Obs *Good quality 8*	BM samples (*n* 100), self-administrated dietary record of the day before milk samples collection every day for 1 week	Not provided	Morning milk, manual expression	Mean (sd) BM from overweight *v*. normal weight women: SFA (46·3 % (4·4) *v*. 43·6 % (6·0), *P* = 0·012), *n*-3 PUFA (2·2 % (0·79) *v*. 2·7 % (1·1), *P* = 0·010), ratio of unsaturated to saturated FA (1·1 (0·2) *v*. 1·3 (0·4), *P* = 0·008). Pearson’s correlation coefficient between the high-fat dairy products and breast milk SFA: 0·21 (0·04).
Olafsdottir, 2006	Healthy lactating women between 2 and 4 months PP Iceland Obs *Fair quality 6*	BM samples (*n* 77), 24-h recalls and food questionnaire on fish consumption and dietary habits. Two groups: women consuming (*n* 18) *v*. not consuming cod liver oil (*n* 59)	Not provided	Collection of four times per d	Proportion of PUFA in the diet is significantly higher among women consuming cod liver oil. It also gives higher % of DHA, EPA and DPA *n*-3 in BM. Correlation coefficient between maternal diet and milk FA composition (% of total FA): maternal PUFA: SFA and milk ALA, 0·336 (*P* = 0·003): maternal PUFA and milk ALA, 0·432 (*P* < 0·001): maternal PUFA and milk EPA, 0·302 (*P* = 0·008): maternal protein and milk EPA, 0·362 (*P* = 0·001): maternal protein and milk DPA, 0·373 (*P* = 0·001): maternal protein and milk DHA, 0·346 (*P* = 0·002)
Perrin, 2019	Healthy lactating women ≥ 2 weeks PP USA Obs *Good quality 7*	Single BM sample from vegan lactating women (*n* 26), vegetarian lactating women (*n* 22) and omnivore lactating women (*n* 26)	Not provided	Sample collected in the morning during first and second breast-feeding of the day and at least 2 h since previous breast-feeding in a dimly lit room to protect light-sensitive nutrients	Vegan, vegetarian and omnivores median (IQR) unsaturated fatty acids were 66·0 % (6·5 %), 57·8 % (9·8 %) and 56·2 % (8·5 %), respectively (*P* < 0·001). Total *n*-3 PUFA were 2·29 % (0·77 %) for vegans 1·55 % (0·56 %) for vegetarians and 1·46 % (0·94) for omnivores (*P* < 0·001), with significant difference driven by higher ALA (*P* < 0·001). Ratio of LA to ALA was significantly lower (*P* < 0·001) in BM vegans 9·3 % (2·1 %) compared with vegetarians 12·2 % (4·9 %) and omnivores 12·7 % (6·2 %). No significant differences in DHA, but over 80 % had levels below 0·30 %. Reports of *n*-3 PUFA supplement and seafood consumption were limited
Sanders, 1978	Vegan and omnivore healthy lactating women between 2 and 6 months PP UK Obs *Fair Quality 6*	BM samples (*n* 8)	Caucasian	Sample collected at the start of the morning	BM of vegans contained lower proportions of 16:0, 16:1, 18:0 and 20:4 *n*-3 and higher proportions of 18:2 *n*-6 (*P* < 0·05)
Sanders, 1992	Healthy lactating mothers < 14 weeks PP UK Obs *Fair quality 6*	Milk samples from (*n* 45); (*n* 19 vegans, *n* 5 vegetarians and *n* 21 omnivores); 3-d food dietary	White vegetarians and vegan, Indian vegetarians and Whit e omnivores	Not provided	In comparisons to omnivores, vegan’s BM contains higher proportion of SCFA (C10–C14) and lower proportion of medium-chain FA (C16–C18); (*P* < 0·01). Same proportion of ARA is in all groups, and proportion of BM DHA is lower in vegans than in omnivores and vegetarians (*P* < 0·01). The *n*-6/*n*-3 FA ratio was higher in the vegan group than in the others
Scopesi, 2001	Healthy lactating women < 1 month PP Italy Obs *Fair quality 6*	BM samples (*n* 34) 1 d PP, 4 d PP, 14 d PP, 21 d PP and 28 d PP. Dietary questionnaire referred to the day prior milk extraction for sampling	Not provided	Not provided	Pearson’s correlation coefficient between maternal dietary intake and corresponding BM concentrations (% of total FA): SFA, 0·60 (*P* < 0·01) in transitional milk; MUFA, 0·63 (*P* < 0·01) in transitional milk; PUFA, 0·65 (*P* < 0·01) in mature milk

RCT, randomised control trial; BM, breast milk; PP, postpartum; AM, ante meridiem ; PM, post meridiem; M, median; GA, gestational age; Obs, observational study; ALA, *α*-linolenic acid; ARA, arachidonic acid; ALA, α-linolenic; LA, linoleic acid; DGLA, dihomo-γ-linolenic acid; DTA, docosatetraenoic acid; GLA, γ-linolenic acid.

**Table 2.** Responsivity of breast milk vitamin content to maternal diet

Ref	Participants Risk of bias	Study	Ethnicity	Breast milk collection timing	Effects on breast milk vitamin content
Vitamin A					
Experimental studies					
Bahl, 2002	Healthy women 18–42 d PP Ghana, India and Peru RCT *Some concerns*	Single dose (60 mg) retinyl palmitate (*n* 322) or control (soyabean oil, *n* 309). Milk retinol levels at 0, 2, 6 and 9 months	Hispanic, Indian and African	Usually collected between 09.00 and 12.00	Significantly higher retinol in treatment group at 2 months only (difference in means 7·1 nmol/g fat, 91 % CI 3·4, 10·8, *P* < 0·05)
Basu, 2003	Healthy lactating women after delivery India RCT *High Risk of bias*	Single dose (209 μmol, 200 000 μg) retinol within 24 h of delivery (*n* 139), and untreated control group (*n* 132). Milk retinol levels at 0 and 24 h, and 1–6 months	Asian	Colostrum collected by manual breast pump, no information in the collection timing	Treatment group had significantly higher retinol levels up to 4 months (*P* < 0·01)
Canfield, 2001	Lactating women 7 months PP Honduras RCT *Some concerns*	90 mg *β*-carotene as red palm oil (*n* 32), *β*-carotene supplements (*n* 36) or placebo (*n* 18). Six doses over 10 d. Milk retinol, lutein, *β*-cryptoxanthin, lycopene, *α*- and *β*-carotene at 0 and 10 d	Hispanic	Mid-morning collection by manual expression	No significant difference in retinol, but palm oil supplementation led to greater increases in lutein, lycopene, *α*- and *β*-carotene *v*. control. Increases in *β*-carotene concentrations were greater for the palm oil group (2·5-fold, *P* < 0·0001) than for the *β*-carotene concentrations supplement group (1·6-fold, *P* < 0·006) relative to placebo
Canfield, 1998	Healthy lactating women < 6 months PP USA RCT *High Risk of bias*	Participants received a single dose of 60 mg (group 1, *n* 6) or 210 mg (group 2, *n* 6) of β-carotene. Milk retinol. α-Tocopherol and carotenoids were monitored for 8 d	Not provided	Mid-afternoon	Data show that a single 60 mg supplement of β-carotene sustained elevated β-carotene concentrations in milk for > 1 week in healthy mothers but did not affect concentrations of other carotenoids, retinol and α-tocopherol β-Carotene concentration in BM group 1 *v*. group 2, mean (se): 36·1(5·5) nmol/g lipid *v*. 50·4 (16·8) nmol/g lipid. α-Carotene concentration in BM group 1 *v*. group 2, mean (se): 10·4 (2·4) nmol/g lipid *v*. 14·0 (5·0) nmol/g lipid Lycopene concentration in BM group 1 *v*. group 2, mean (s e): 18·7 (3·4) nmol/g lipid *v*. 34·7 (5·6) nmol/g lipid (*P* < 0·05)
Ding, 2021	Healthy lactating women 30–45 d PP China RCT *Low risk of bias*	Supplementation for 2 months with 1800 μg vitamin A and 600 μg vitamin D (*n* 117), or placebo (*n* 128)	Asian	BM collected between 07.30 and 09.00 through breast pump	After 2 months vitamin A in supplemented group was M = 1 (sd = 0·5) µmol/l *v*. control group M = 0·8 (sd = 0·5) µmol/l, *P* < 0·05.
Gossage, 2002	Healthy lactating women < 32 d PP USA RCT *Some concerns*	Subjects (*n* 21) received 30 mg/d of β-carotene or placebo from day 4 to day 32 PP. BM samples analysed for concentrations of carotenoids, α-tocopherol, and retinol. Eight diet records and eight BM samples	Two African American, two Hispanic and 17 European American	Not provided	No significant effects of β-carotene supplementation on BM concentrations of lutein, β-cryptoxanthin, lycopene or α-carotene. Milk concentrations for all four carotenoids decreased over time (*P* < 0·01 for all). Milk concentration in retinol and*α*- tocopherol were unaffected by supplementation and decreased over time (*P* < 0·0050. Mean (sd) retinol concentration were 4944 (539) µmol/l initially and 2079 (207) µmol/l at the end of the study. α-Tocopherol concentrations were 31 (4·6) µmol/l initially and 9·4 (1·2) µmol/l at the end of the study
Grilo, 2016	Healthy puerperal women 24 h PP Brazil RCT *Some concerns*	Single dose (60 mg, 200 000 μg) retinyl palmitate after first colostrum collection (*n* 30), and untreated control group (*n* 27). Milk retinol and *α*-tocopherol levels at 0 and 24 h and 30 d	Not provided	Collection after an overnight fast from 08.00 until 12.00	Intervention significantly increased retinol levels at 24 h (+157 %, *P* < 0·001); however, retinol levels of both groups did not differ at 30 d. After supplementation, colostrum *α*-tocopherol decreased significantly (–16·4 %, *P* < 0·05), but no significant difference in *α*-tocopherol levels between groups at 30 d.
Johnson, 1997	Healthy lactating women < 8 months PP USA RCT *High Risk of bias*	Subjects given either placebo (*n* 4) or naturally occurring β-carotene (64 mg all-trans BC and 69 mg 9-cis BC; *n* 8) for 8 d	Not provided	Not provided	For supplemented group, significant increase in concentration observed at day 3 (*P* < 0·001) and steadily increased to six times the baseline level by the end of the supplementation period (day 8, *P* < 0·001). After 1 month, BM concentration of all-trans BC decreased but was still significantly higher than day 1 (*P* < 0·022).
B vitamins					
Experimental studies					
Chang, 2002	Healthy lactating women < 1 month PP USA RCT *High risk of bias*	Four groups of lactating mothers (*n* 47) receiving 2·5, 4, 7·5 and 10 mg/d of PN-HCl, 24 h dietary record.	Not provided	Not provided	BM vitamin B_6_ responsive to maternal supplementation from 1–6 months PP. Mean BM B_6_ significantly lower for women supplemented with 2·5 mg PN-HCl/d than for those supplemented with 4·0, 7·5 or 10·0 mg/d. Mean (sem) range from 1–6 months for groups supplemented with 2·5 mg/d (891 (29·9) to 1·316 (74·8) nmol/l), 4 mg/d (1184 (40·8) to 1944 (74·8)), 7·5 mg/d (1·752 (86·7) to 2278 (86·7)), and 10 mg/d (1704·3 (38·9) to 2338·1 (104·7) nmol/l).
Hampel, 2017	Healthy lactating women 2–4 months PP Bangladesh RCT Low risk of bias	3-d supplementation study (*n* 18). Day 1: fasting no supplement, day 2: one time the US Canadian RDA for vitamins, day 3: Twice US Canadian RDA for vitamins. BM vitamin A, B_1_, B_2_, B_3_, B_6_, B_12_ and E measured	Not provided	Breast milk collected at each feeding through a breast pump form the same breast for 24 h	BM vitamin A, B_1_, B_2_ and B_6_ significantly increased in BM-supplemented group, median increases > 180 % (B_2_ and B_6_) and 120–130 % for B_1_ and A. B_3_ and E levels significantly lower on days supplements were consumed (*P* < 0·05). No significant effect of supplementation for B_12_
Nail, 1980	Healthy lactating women at parturition USA RCT *Some concerns*	Two groups, one (*n* 7) received vitamin B_1_ and B_2_ supplementation (B_1_ 1·7 mg/d, B_2_ 2·0 mg/d), second group (*n* 5) received no supplementation. BM samples after 1 and 6 weeks supplementation	Not provided	Not provided	BM mean (sd) B_1_ of both groups after 1 and 6 weeks, respectively were: non-supplemented 138 (18) µg/l and 220 (27) µg/l; and supplemented 133 (27) µg/l and 238 (21) µg/l. There were significant increases in B_1_ both groups (*P* < 0·05). BM mean (sd) B_2_ of both groups after 1 and 6 weeks, respectively, were non-supplemented 367 (128) µg/l and 485 (123) µg/l and supplemented 880 (168) µg/l and 710 (187) µg/l
Styslinger, 1985	Healthy lactating women 2–3 months PP USA RCT *High risk of bias*	0, 2·5, 10·0 or 20·0 mg pyridoxine-HCl for three consecutive days in addition to dietary sources (*n* 6). BM samples at baseline and 3 d	Not provided	Not provided	Significant positive correlation (*r *= 0·80, *P* < 0·001) between supplemental intake and vitamin B_6_ content
Thomas, 1979	Healthy lactating women 1 week PP USA RCT *High risk of bias*	No supplement (*n* 7), or multivitamin and multimineral supplement (*n* 10), containing 4 mg vitamin B_6_. BM measured for 3 d periods at 1 and 6 weeks	Caucasian	Milk samples expressed four times a day at 4 h interval	Vitamin B_6_ differed significantly (*P* < 0·05) at 5–7 d. At 43–45 d, content significantly increased (*P* < 0·05) in supplemented group. Content in supplemented group remained constant at 43–45 d, and the difference between groups at 43–45 d was not significant
Thomas, 1979	Healthy lactating women 1 week PP USA RCT *High risk of bias*	Non-supplement (*n* 7) and multivitamin and multimineral supplement (*n* 10), containing 8 μg of vitamin B_12_. BM measured for 3 d periods at 1 and 6 weeks	Caucasian	Milk samples expressed four times a day at 4 h interval	No significant differences between the groups at 5–7 d. Decline in both groups at 43–45 d. However, levels in non-supplemented group were significantly lower than both the 5 to 7 d levels. Mean (sd) values for non-supplemented were 1·22 (0·41) µg/l at 5–7 d and 0·61 (0·17) µg/l at 43–45 d, (*P* < 0·05), and supplemented group at 43–45 d PP; and supplemented were 1·65 (0·63) µg/l at 5–7 d, 1·10 (0·57) µg/l, (*P* < 0·05).
Vitamin C					
Experimental studies					
Byerley, 1985	Healthy lactating women 11 weeks PP Canada RCT *High risk of bias*	Five vitamin C groups (1) 0 mg, 0 mg 1 d + 90 mg per for 2 d, (3) 90 mg for 1 d + 250 mg per d for 2 d, (4) 90 mg for 1 d + 500 mg per d for 2 d, (5) 90 mg for 1 d + 1000 mg per d for 2 d, all *n* 5	Not provided	BM collected at each feeding either manually or by use of breast pump	Mean vitamin C ranged from 44 to 158 mg/l but not correlated with intake and not significantly different between groups
Daneel Otterbech, 2005	Healthy lactating women Switzerland and Republic of Ivory Coast 8 mPP RCT *High risk of bias*	Five separate studies: 1. Baseline milk ascorbic acid levels European (*n* 142) and African (*n* 171) women. 2. 1000 mg ascorbic acid per d for 10 d, European (*n* 10) and African (*n* 18) women. 3. European women (*n* 17), 1000 mg ascorbic acid per d for 5 d, followed for 35 d. 4. African women (*n* 11), 100 mg ascorbic acid per d for 10 d. 5. 1, 3 or 5 servings of orange juice (100 mg ascorbic acid/serving) per week for six weeks, African women (*n* 15)	Caucasian and African	All samples collected between 07.00 and 12.00	BM vitamin C 50 % lower (*P* < 0·001) from African women. Supplementation (1000 mg/d for 10 d) increased levels in both African and European women, from 19 to 60 mg/kg (*P* < 0·001) and 60 to 70 mg/kg (*P* < 0·03), respectively
Thomas, 1979	Healthy lactating women 1 week PP USA RCT *High risk of bias*	Non-supplement (*n* 7) and multivitamin and multimineral supplement (*n* 10), containing 90 mg vitamin C from parturition. BM analysed for 3 d periods at 1 and 6 weeks	Caucasian	BM samples expressed four times a day at 4 h intervals	No significant differences in levels between groups at any time points
Vitamin D					
Experimental studies					
Ala-Houhala, 1988	Healthy lactating women 8 weeks and 20 weeks PP USA RCT *High risk of bias*	Daily supplementation for 8 weeks (Winter) and 15 weeks (Spring) with 2000 μg (*n* 15) vitamin D, 1000 μg vitamin D (*n* 15), or no supplementation (*n* 15)	Not provided	Morning milk	Oral maternal supplementation of vitamin D had no significant effect on milk vitamin D levels, but 25(OH) D levels of mothers receiving either 1000 or 2000 μg (25 or 50 micrograms) vit D/d were significantly higher than those of non-supplemented mothers in February and April
Basile, 2006	Healthy lactating women 1 month PP USA RCT *Low risk of bias*	Subjects received (*n* 12) 2000 μg/d vitamin D or (*n* 13) 4000 μg/d for 3 months. BM samples collected to measure vitamin D	16 White and 9 Africa*n*-American	Not provided	25 (OH) D increased from 1 to 4 months in both group (mean (sd)): (+11·5 (2·3)) ng/ml for group 2000 (*P* = 0·002) and (+14·4 (3·0)) ng/ml for group 4000 (*P* = 0·0008). The 4000 μg/d regimen was more effective in raising BM antirachitic activity than the 2000 μg/d supplementation. Decline in BM was not associated with vitamin D dose (*P* = 0·73) or maternal 25(OH)D (*P* = 0·94)
Ketha, 2018	Healthy lactating women < 6 months PP USA RCT *Low risk of bias*	Subjects (*n* 40) received either a single dose 150 000 μg or 5000 μg daily of vitamin D3 for 28 d. BM vitamin D measured at 1, 3, 7, 14 and 28 d. Outcome was the temporal changes in 24,25 (OH)2D3/25(OH)D3 ratio	Not provided	Not provided	BM vitamin D3 values in the single-dose group were inversely associated with 24,25 (OH)2D3/25(OH)D3 ratio (r2 = 0·14, *P* < 0·001), but not with daily dosing
Niramitmahapanya, 2017	Healthy lactating women < 6 weeks PP Thailand RCT *Low risk of bias*	Subjects received either 800 μg/d vitamin D supplement (*n* 35) for 6 weeks or a placebo (*n* 33)	Asian	Not provided	BM vitamin D at baseline mean (sd) supplemented group *v*. non-supplemented group: 79·86 (18·27) nmol/l *v*. 88·33 (21·28) nmol/l, (*P* = 0·183). Vitamin D BM concentration at 6 weeks supplemented group *v*. non-supplemented group: 97·49 (19·32) nmol/l *v*. 88·92 (22·42) nmol/l, (*P* = 0·076)
Oberhelman, 2013	Healthy lactating women < 6 months PP USA RCT *Some concerns*	Single dose 150 000 μg cholecalciferol (*n* 20) or 5000 μg/d cholecalciferol (*n* 20) for 28 d. BM cholecalciferol and 25(OH)D measured on 0, 1, 3, 7, 14 and 28 d	Not provided	Not provided	BM mean cholecalciferol reached peak of 40 ng/ml at 1 d in single-dose group, whereas in the daily supplemented group levels remained at approximately 8 ng/ml from 3 to 28 d
Vitamin E					
Experimental studies					
Clemente, 2015	Healthy lactating women 12 h PP Brazil RCT *Low risk of bias*	Non-supplemented control (*n* 36), single dose 400 μg RRR-*α*-tocopherol (*n* 40), or single dose 400 μg all-rac-*α*-tocopherol synthetic (*n* 33). BM colostrum *α*-tocopherol measured 12 h PP and 24 h after supplementation	Not provided	Colostrum collected by manual expression, no information on timing	No change in control group at 24 h, whereas RRR *α*-tocopherol and all-rac *α*-tocopherol increased by 57 % and 39 %, respectively (significantly different between control group and *α*-tocopherol synthetic group and control group and *α*-tocopherol natural group, *P* < 0·001); significantly different between *α*-tocopherol synthetic group and *α*-tocopherol natural group, *P* = 0·04)
Gaur, 2017	Healthy lactating mothers < 4–6 weeks PP USA RCT *Low risk of bias*	3 groups (*n* 89), group 1 (*n* 29) received 45·5 mg all-rac-*α*-tocopherol acetate, group 2 (*n* 30) 22·8 mg all-rac-α-tocopherol acetate + 20·1 mg RRR-*α*-tocopherol, group 3 (*n* 30) 40·2 mg RRR α-tocopherol for 6 weeks	Not provided	Not provided	In group 3, % of RRR-*α*-tocopherol increased in BM (mean (sem): 78 % (2·3 %) compared with 82 % (1·7 %) (*P* < 0·05). In contrast, the % of RRR-α-tocopherol decreased in the group 2 (*P* < 0·05) and group 1 (*P* < 0·0001)
Kanno, 1989	Healthy lactating mother 70 d parturition Japan RCT *High risk of bias*	d-α-Tocopherol (1·1 g) in a capsule was orally administrated once with ice cream to a mother	Not provided	BM expressed 2–3 times daily with manual pump	The transfer of α-tocopherol into BM reached a maximum value of 414 µmol/100 g after 3 d and then declined to the baseline level after 5 d. The amount of α-tocopherol recovered in BM was 0·11 %. The *α*-tocopherol equivalent/PUFA ratio (mg/g) was increased from 0·25 to value between 0·7 and 1·7
Pires Medeiros, 2016	Healthy lactating women < 30 d PP Brazil RCT *High risk of bias*	Non-supplemented control (*n* 51), of single dose 400 μg RRR-*α*-tocopherol (*n* 38). BM *α*-tocopherol measured after delivery, 24 h, 7 and 30 d	Not provided	Samples collected after an overnight fast	BM *α*-tocopherol increased by 60 %, 24 h after, but not control group (*P* < 0·001). At 7 d supplemented group levels 35 % higher than control group, but no difference at 30 d
De Souca Reboucas, 2019	Healthy lactating women 30–90 d PP Brazil RCT *Some concerns*	Single dose 800 μg (588 mg, RRR-*α*-tocopherol, (*n* 39), or non-supplemented control (*n* 40). BM *α*-tocopherol measured at supplementation and next day.	Not provided	BM collected manually from a single breast that had not been collected previously	No difference between control and supplemented groups at baseline. One day after supplementation, supplemented group levels significantly increased by 124 % (mean = 15·00 µmol/l, sd = 5·1 µmol/l, *P* < 0·001), with no change in control group (mean = 6·94 µmol/l, sd = 2·0 µmol/l)
Observational studies					
Antonakou, 2011	Healthy lactating mothers < 1 month PP Greece Obs *Good quality 8*	BM samples (*n* 64), 3 d dietary record at 1, 3 and 6 months PP	Not provided	Morning hour BM collection by electric pump	BM mean (sd)*α* tocopherol was 8·3 (3·4) µmol/l, 8·1 (4·2) µmol/l and 8·5 (4·7) µmol/l at 1, 3 and 6 months PP, respectively; while total tocopherol values were 8·9 (3·6) µmol/l, 8·7 (4·6) µmol/l and 9·5 (5·6) µmol/l, respectively. No significant differences observed between the time points. Mean (sd) maternal vitamin E dietary intake was 7·2 (3·7) mg/d, 6·8 (3·5) mg/d and 10·9 (5·2) mg/d at 1, 3 and 6 months PP, respectively. Though, vitamin E dietary intake was lower than the recommended one. Correlation of dietary intake parameters with the concentration of vitamin E in mature milk at first month of lactation: total fat (% total fat), *r *= 0·244, *P* = 0·047, PUFA (% total fat) *r *= 0·092, P = 0·387; MUFA (% total fat) *r *= 0·195, *P* = 0·062
Vitamin K1					
Experimental studies					
Bolisetty, 1998	Healthy lactating women with preterm births, 28–32 weeks RCT *High risk of bias*	2·5 mg phylloquinone (vitamin K1) daily for 2 weeks (*n* 6). BM phylloquinone measured daily for 14 d	4 Caucasian and 2 Asian	BM extracted either manually or with electric pump at 5 h intervals	Mean (sd) BM levels increased from baseline: 3 (2·3) ng/ml to 22·6 (16·3) ng/ml, (*P* < 0·05) after the first dose, with continual increase until plateau at 64·2 (31·4) ng/ml after sixth day
Greer, 1997	Healthy lactating mothers < 3 d PP USA RCT *Low risk of bias*	2 groups: either 5 mg of phylloquinone (*n* 11) or placebo (*n* 11), daily supplementation for 12 weeks. Placebo was glucose. BM collected after 2 weeks, 6 weeks and 12 weeks supplementation	Not provided	Not provided	Mean (sd) BM vitamin K supplemented *v*. placebo: 1·10 (0·75) ng/ml *v*. 0·69 (0·39) ng/ml at baseline; 76·53 (26·98) ng/ml *v*. 1·17 (0·70) ng/ml after 2 weeks (*P* < 0·01); 75·27 (46·23) ng/ml *v*. 1·14 (0·46) ng/ml after 6 weeks (*P* < 0·01)
Von Kries, 1987	1 lactating mother Germany < 5 weeks PP RCT *High risk of bias*	Single dose of 0·5 mg, 1 mg and 3 mg vit K1 (*n* 1 per group). Dose response (0·1 mg, 0·5 mg, 1 mg and 3 mg) measured over 24 h (*n* 1). Single dose study measured BM vitamin K1 over 50 h, and dose–response study measured over 24 h	Not provided	Complete expression of both breasts using an electric pump	0·5 mg, 1 mg and 3 mg produced increases in milk levels, peaking at 12–24 h. Dose–response relationship observed, with the lowest dose (0·1 mg) producing 2-fold increase in vitamin K1 content. 100 µg dose of vitamin K1 raised BM vitamin K1 from 2·5 to 4·9 ng/ml after 16 h, this then declined to 1·9 ng/ml after 24 h

PP, postpartum; RCT, randomised control trial; BM, breast milk; AM, ante meridiem; PM, post meridiem; M, median; Obs, observational study.

**Table 3.** Responsivity of breast milk mineral, amino acid and protein content to maternal diet

Ref	Participants Risk of Bias	Study	Ethnicity	Breast milk collection timing	Effects on breast milk content
Iodine					
Experimental studies					
Leung, 2012	Healthy lactating women over 3 months PP USA RCT *High risk of bias*	600 µg oral potassium iodide (456 µg iodine) after overnight fast (*n* 16). Iodine measured in BM at baseline and hourly for 8 h after intake and dietary iodine recorded	Not provided	Not provided	Following supplementation, there was a significant median increase in BM iodine levels (280·5 μg/l; IQR 71·5–338·0) above baseline (*P* < 0·01); the median peak iodine was 354 μg/l (IQR 315–495)
Mulrine, 2010	Healthy lactating women after delivery New Zealand RCT *Low risk of bias*	Placebo (*n* 56), 75 µg iodine/d (*n* 27) or 150 µg iodine per d (*n* 26) for 24 weeks. BM iodine measured at 1, 4, 8, 12, 16, 20 and 24 weeks	Most likely White Caucasian	All BM samples collected from 09.00 and 12.00	Iodine decreased by 40 % over 24 weeks in placebo group (*P* < 0·001) but was 1·3 times higher in 75 µg supplemented group (*P* = 0·003) and 1·7 times higher in 150 µg supplemented group (*P* < 0·001)
Nazeri, 2016	Healthy lactating mothers 3–5 d PP Iran RCT *Low risk of bias*	Either 150 µg iodine/d (*n* 42), or no supplementation, but recommendation to used only iodised salt for cooking (control) (*n* 42). Iodine measured at 0, 7, 10, 14 and 30 d	Persian	Manual expression but no information on timing	At baseline: Median (IQR) 176·0 µg/l (133·7–218·7 µg/l) in supplemented group and 215 µg/l (168·5–315·5 µg/l) in control group, (*P* = 0·027). d7:191·0 µg/l (105·0–245·0) in supplemented group and 176·0 µg/l (140·0–286·0 µg/l) in control; d10:217·0 µg/l (148·7–339·0 µg/l) in supplemented group and 162·0 µg/l (120·0–206·5 µg/l) in control; d14:242·0 µg/l (156·2–355·7 µg/l) supplemented group and 160·0 µg/l (115·2–199·2 µg/l) in control; d30:210·0 µg/l (100·0–286·0 µg/l) supplemented group and 142·0 µg/l (92·2–197·2 µg/l) in control
Observational study					
Ureta-Velasco	Milk donors Spain Obs Good quality 8	BM iodine level from milk donors (*n* 113) measured and analysed together with five dietary records	Caucasian	Not provided	Iodine positively correlated with total iodine intake (rho = 0·0499, *P* < 0·001), but not iodine intake from food only (rho = 0·046, *P* = 0·628). Iodine positively correlated with consumption of > three dairy products per d
Se					
Experimental studies					
Dodge, 1999	Healthy lactating women from delivery New Zeeland RCT *Some concerns*	50 µg Se per d (*n* 12), or placebo (*n* 10) during pregnancy and lactation for 3 months.	Caucasian	First morning milk was collected using a breast pump	Se increased by 37 % (*P* = 0·003) following supplementation, glutathione activity was unchanged. Supplementation increased PUFA levels by 41 %; (placebo: mean = 9·7, sd = 1·24, *v*. supplemented: mean = 13·70, sd = 1·02), including LA and ARA, all *P *≤ 0·05. SFA levels were correspondingly decreased by 11 % (placebo: mean = 50·1, sd 2·0, *v*. supplemented: mean = 44·4, sd = 1·6 g/100, *P *≤ 0·04). Fatty acid values all expressed as g/100 g fatty acids
Dylewski, 2002	Healthy lactating women 3 months PP USA RCT *High risk of bias*	20 µg Se per d for 3 months (*n* 23). Dietary data and milk at 3 and 6 months	Not provided	Not provided	BM mean (sd) Se dietary intake was 111 (40) µg/d and did not change over the study. Supplementation significantly increased levels by 41 % from 3 (23 (7) ng/ml) to 6 months (32 (14) ng/ml), *P *≤ 0·01
Trafikowska, 1996	Healthy lactating women 3–4 weeks PP Poland RCT *High risk of bias*	Subjects (*n* 16) supplemented with 200 µg/d Se in the form of yeast-rich-Se for 3 months	Not provided	Not provided	After 1 month of Se supplementation, the Se concentration in milk increased significantly (*P* < 0·001) by 73 % to a plateau of 14–16 ng/ml
Trafikowska, 1998	Healthy lactating women 3–5 weeks PP Poland RCT *High risk of bias*	3 groups; group 1 (*n* 24) supplemented with 200 µg/d Se (yeast-rich Se, group 2 (*n* 30) supplemented with 200 µg/d selenite mixed with baker’s yeast, group 3 (*n* 13) supplemented with plain brewer’s yeast without Se. Supplementation lasted 3 months	Not provided	BM collected by manual expression prior to the first morning feeding	Baseline BM (sd) Se 8·9 (2·8) µg/l. In the control group, it remained constant during the 3-month period. Group 1 and 2; BM Se increased significantly reaching a plateau of 14–16 µg/l after 1 month of supplementation. The difference was significantly higher than controls in the yeast-rich Se (*P* < 0·001) and the selenite-Se-supplemented group (*P* < 0·01)
Observational studies					
Bianchi, 1999	Healthy lactating mothers < 210 d PP Brazil Obs *Fair quality 6*	BM from (*n* 30) mothers, 24-h recall FFQ. BM collected at 7 d and 270 d. BM Se analysed	Nor provided	Not provided	No significant correlation between BM Se and maternal BMI (r2 = −0·0654, *P* = 0·7351); Se intake (r2 = −0·103, *P* = 0·594) and stage of lactation (r2 = −0·2981, *P* = 0·1095)
Valent, 2011	Healthy lactating mothers 3 months PP Italy Obs *Good quality 8*	BM from (*n* 100) mothers, semi-structured dietary questionnaires. BM Se analysed.	Not provided	Milk collected at any time	BM Se significantly correlated with current fresh fish consumption, *r *= 0·21, *P* = 0·04. No significant correlation between intake of multivitamin supplements during pregnancy and Se in BM (mean 10·9 (3·3) ng/g, median 9·6 *v*. mean 12·3 (2·9) ng/g. median 11·4 for women who did not consume supplement, *P* = 0·11)
Zn/Cu/Fe					
Choi, 2016	Healthy women 5–15 d PP Korea Obs *Good quality 9*	*n* 79 participants completed 3 d dietary record.	Asian	Not provided	Mean (sd) Fe significantly higher in BM from those taking daily Fe supplements (7·36 (9·10) mg/ml, *n* 64) *v*. those not (2·83 (6·36) mg/l, *n* 15), (*P* = 0·002). No significant difference from those taking daily Zn supplementation (0·36 (0·18) mg/l, *n* 64) *v*. those not (0·40 (0·17) mg/l, *n* 15). No significant difference from those taking daily Cu supplementation (0·69 (0·27) mg/l, *n* 64) *v*. those not (0·70 (0·22) mg/l, *n* 15)
Leotsinidis, 2005	Healthy lactating mothers 3 d PP Greece Obs *Fair quality 5*	BM samples collected (*n* 180) to measure Cd, Cu, Fe, Pb, Mn and Zn at 3 and 14 d PP, FFQ	Not provided	Morning BM 2 h after previous breast-feeding	Mean (sd) values of colostrum samples: Cd, 0·190 (0·150) µg/l; Cu, 381 (132) µg/l; Fe, 544 (348) µg/l; Pb, 0·48 (0·60) µg/l; Mn, 4·79 (3,23) µg/l; Zn, 4905 (1725) µg/l. All metals with exception of Cu were found in lower concentrations in transitory milk
Vuori, 1980	Healthy lactating mothers 6–8 weeks PP Finland Obs *Fair quality 6*	BM samples collected to measure Fe and Zn at 6–8 weeks PP and 17–22 weeks PP, 7 d FFQ *n* 15	Not provided	BM collected at beginning and end of each breast-feeding	Correlation coefficient (r) between enery intake (Kcal) and BM minerals (mg): Fe, 0·478 (*P* < 0·01) and Zn, 0·554 (*P* < 0·01)
Fe					
Experimental studies					
Yalcin, 2009	Healthy lactating women 2 weeks PP Turkey RCT *Low risk of bias*	Placebo (*n* 23) or 80 mg/d Fe (*n* 24) for 16 weeks. BM Fe and Zn at baseline, and Fe at 2 and 16 weeks	Turkish	Collection in morning before feeding infant, 2 h after previous breast-feeding	Fe supplementation to non-anaemic women did not change BM Fe content. BM mean (sd) supplemented Fe concentration was 579 (219) µg/l in week 2 and 372 (163) µg/l in week 16; whereas placebo 512 (178) µg/l week 2, and 385 (207) µg/l, in week 16
Tyrosine					
Experimental studies					
Downlati, 2014	Healthy lactating women from 2 to 24 months PP Canada RCT *Some concerns*	Single dose 0, 2, 5 and 10 g oral tyrosine (*n* 24). Free and total tyrosine measured before and 2, 4 and 6 h after supplementation	Not provided	Not provided	Significant rise only in free tyrosine. 10 g of tyrosine group had significantly higher free tyrosine concentration compared with other groups (*P* < 0·001). Peak free tyrosine in BM after 10 g dose occurred at 4 h, whereas for the 2 g and 5 g tyrosine doses, maximum free tyrosine levels occurred at 6 h
Protein, amino acids, ovalbumin					
Experimental studies					
Forsum, 1980	Healthy lactating women 13–20 weeks PP Sweden RCT *Some concerns*	4 d of low protein diet, 1 d wash out and then 4 d of high-protein diet (*n* 3)	Not provided	Milk collected before and after each nursing by hand expression or manual pump	Mean (sd) protein content in BM (g/d) for low protein diet *v*. high protein diet: true protein 7·31 (0·74) *v*. 8·83 (0·44) (*P* < 0·05); lactoferrin (g/24 h), 2·52 (0·17) *v*. 3·01 (0·36); *α*-lactalbumin (g/24 h) 1·50 (0·20) *v*. 1·75 (0·12); lactose (g/24 h), 58·1 (13·2) *v*. 63·5 (5·6). Differences in lactoferrin, α-lactalbumin and lactose between the two groups not significant
Metcalfe, 2016	Healthy lactating women first 6 weeks PP Australia RCT *High Risk of Bias*	Groups comprised high-egg diet (> 4 eggs per week, *n* 40), low-egg diet (1–3 egg per week, *n* 44), and egg-free diet (*n* 36). BM measured at 2, 4 and 6 weeks.	85 % Caucasian	BM collected between 2 and 6 h after previous breast-feeding	Mean egg consumption associated with ovalbumin concentration, whereby each additional egg ingested per week led to 25 % increase in ovalbumin levels (95 % CI 5, 48 %, *P* = 0·01). Ovalbumin significantly higher in high egg group compared with egg-free group. One third of women had no ovalbumin detected
Palmer, 2016	Healthy lactating women 11–14 weeks PP Australia RCT *Some concerns*	Groups comprised, no egg, one raw egg, half a cooked egg and one cooked egg (all *n* 41). BM samples collected every 2 h for 8 h.	Not provided	Not provided	Direct dose–response between amount of cooked egg ingested and peak of ovalbumin in BM (no egg 0·05 ng/ml (95 % CI 0·01, 0·1), half a cooked egg 2·24 ng/ml (95 % CI 0·57, 3·91), one cooked egg 3·16 ng/ml (95 % CI 1·41, 4·91), *P* < 0·05. No difference between raw and cooked eggs. No ovalbumin detected in BM of 24 % of women
Observational studies					
Rana, 1986	Healthy lactating women 4–6 weeks PP England Obs *Poor quality 2*	Group 1 followed vegan diet for a year, no medication, no contraceptives (*n* 14), group 2, control (*n* 14) omnivore diet. FFQ 7 consecutive d	Not provided	Not provided	Mean taurine concentration in the vegan BM 35 mg/l was significantly lower than in BM of omnivores (55 mg/l) (*P* < 0·01)
Choline					
Observational studies					
Perrin, 2020	Healthy lactating women > 2 weeks PP USA Obs *Fair quality 7*	FFQ to classify as non-vegetarian, vegetarian or vegan (*n* 74). Single BM sample measured free choline, PC and GPC.	Not provided	Samples collected in morning during first or second breast-feeding of day and last 2 h after previous breast-feeding	Wide range in free choline (4–301 mg/l), with no significant differences between groups. Significantly higher GPC in vegan (mean = 62·7 mg/l, sd = 25·3 mg/l) than vegetarian (mean = 47·7 mg/l, sd = 21·2 mg/l) and non-vegetarian (mean = 42·4 mg/l, sd = 14·2 mg/l), *P* = 0·005. Significantly lower PC in vegan (mean = 32·5 %, sd = 18·3 %) than vegetarian (M = 46·1 %, sd = 18·3 %) and non-vegetarian (Mean = 44·8 %, sd = 15·7 %), *P* = 0·01.

RCT, randomised control trial; PP, postpartum; BM, breast milk, obs, observational study; IQR, interquartile range; GPC; glycerol-phosphocholine; PC, phosphocholine.

**Table 4.** Responsivity of breast milk contaminant levels in response to maternal diet

Ref	Participants Risk of bias	Study	Ethnicity	Breast milk collection timing	Effects on breast milk content
Observational studies					
Castro, 2014	Lactating mothers Chile, 2–4 months PP Obs *Fair quality 7*	Participants from Arica (*n* 24) and southern Santiago (*n* 11) completed questionnaires on health status, food habits and breast-feeding practices. BM As, B, Cd, caesium, Ca, Fe, Pb, Li, Mn, Se, U, V and Zn measured	Not provided	BM collected during the morning	No difference in essential and toxic element levels between groups, except B (*P* < 0·001) and Li (*P* < 0·05), which were higher in Arica. Caesium was higher in Santiago samples (*P* < 0·05). Pb positively associated with Fe and Zn (*r *= 0·60, *r *= 0·61, respectively, both *P* < 0·05), but inversely associated with Ca (*r *= −0·61, *P* < 0·05). As associated with Fe (*r *= 0·6, *P* < 0·05), and B and Ca (*r *= 0·54, *P* < 0·05)
Dewailly, 1994	Lactating healthy Inuit women < 3 d PP Artic Quebec Obs *Poor quality 2*	Inuit (*n* 109) participants. Seven PCB and chlorinated pesticides determined in BM and compared with Quebec Caucasian women.	Inuit	Not provided	Mean levels of the different analysed PCB were 3–7 times higher in BM from Inuit *v*. Caucasian women. Seafood consumption was extremely high for Inuit women, at 300 g/d compared with 12 g/d for Caucasian women
Gundacker, 2002	Healthy lactating mothers < 1 month PP Austria Obs *Good quality 8*	Urban (*n* 59) and rural (*n* 47) participants completed questionnaire about dietary habits, smoking, living area and dental filings.	Not provided	Not provided	Low Hg and Pb concentrations were far below currently recommended safety limits (Hg: mean = 1·59 g/l, sd = 1·21 g/l, *n* 116; Pb: mean = 1·63 g/l, sd = 1·66 g/l, *n* 138). Hg was significantly associated with area of residence, prematurity, cereal consumption and vitamin supplementation, whereas for Pb it was area of residence, fish consumption and smoking, all *P* < 0·05
Leotsinidis, 2005	Healthy lactating mothers 3 d PP Greece Obs *Fair quality 6*	BM samples collected (*n* 180) to measure Cd and Pb at 3 and 14 d PP, FFQ	Not provided	Morning milk 2 h after previous breast-feeding	Mean (sd) values of colostrum samples: Cd, 0·190 (0·150) µg/l; Pb, 0·48 (0·60) µg/l. Higher BM lead in samples from urban areas, dietary habits seem to play a role in metal levels in human milk
Ursinyova, 2018	Healthy lactating mothers 6 weeks PP Slovakia Obs *Good quality 7*	*n* 142 participants asked about dietary habits, including frequency and amount of fish consumption per d	Not provided	Hand expression, no information on timing	Total Hg mean = 0·376 µg/l, sd = 0·475 µg/l, with no difference between fish and non-fish eaters. Negative association between BM total Hg and freshwater fish consumption (*β *= −0·193, *P* = 0·017)

Obs, observational study; PP, postpartum; BM, breast milk; PCB, polychlorinated biphenyls.

### Ethnicity

The articles included in this review involved African, Arabic, Asian, Australian, European and Hispanic participants, as summarised in Tables 1–4.

### Main results

#### Fatty acids

Twenty-nine publications on fatty acids were included, fifteen experimental studies^(19–33)^, and fourteen observational studies^(34–47)^ and are summarised in Table 1.

Of the experimental studies, three were rated low risk of bias^(22,25,29)^ and twelve were identified having some concerns^(19–21,23,24,26–29,31–33)^. For the observational studies, three were of good quality^(41,42,44)^, nine were fair quality^(34–36,39,40,43,45–47)^ and two were poor quality^(38,39)^.

#### PUFA

Nine experimental studies were identified with DHA and EPA, and participants were supplemented with DHA in the range of 200 to 1200 mg/d, and EPA between 70 and 300 mg/d^(19,20,22–25,29,31,33)^. DHA and EPA supplementation was consistently shown to increase DHA and EPA breast milk levels, and this was in a dose-dependent manner. Two RCT investigated the effects of maternal *α*-linolenic acid (ALA) and LA maternal supplementation on breast milk, and ALA maternal intake was similarly show to increase breast milk ALA levels^(25,32)^. The observational studies also reported a significant positive correlation between maternal consumption of fatty fish intake and breast milk DHA, EPA and ALA^(36,38,40,43,44)^. The effect of vegan, vegetarian or omnivore diet patterns on breast milk fatty acids fat composition was investigated in three observational studies^(44–46)^. DHA levels were either significantly lower in vegans than omnivores or vegetarians^(46)^ or low across all groups^(44)^. The LA to ALA ratio was significantly lower in breast milk from vegan participants compared with vegetarians and omnivores^(44)^. In comparison to omnivores, breast milk from vegans contains a higher proportion of SCFA (C10–C14) and lower proportion of medium-chain fatty acid (C16–C18). For ARA, only one study was identified, and in this experimental study participants were supplemented with 54 mg ARA per d for 2 weeks and no relationship was identified between maternal intake and breast milk levels^(29)^.

#### Others fatty acids

*Trans*-fatty acids, SFA and hydrogenated fats consumption and their content in breast milk were investigated in four studies^(21,34,37,42)^. The consumption of hydrogenated vegetable oils with high content of *trans*-fatty acid increased the *trans*-fatty acids concentration in breast milk after a 12–36-h lag period.

### Lipid-soluble vitamins (A, D, E and K)

#### Vitamin A

Eight publications were included for vitamin A, which were all experimental studies^(48–55)^. One was low risk of bias^(52)^, four presented some concerns^(48,51,53,54)^ and three were high risk of bias^(49,50,55)^, with the results summarised in Table 2.

Maternal β-carotene supplementation increased β-carotene concentration in breast milk without impacting retinol, *α*-tocopherol or other carotenoid breast milk content. A similar effect is observed with retinol, lactating mothers supplemented with retinol produce a higher retinol concentration breast milk without affecting other carotenoids. The supplements in the experimental studies varied from 30 mg of β-carotene daily to 60 mg of retinyl palmitate or β-carotene single dose and 90 mg β-carotene as red palm oil in six doses over 10 d.

#### Vitamin D

Five publications were included for vitamin D, all experimental studies^(56–60)^ Three were low risk of bias^(57–59)^, one with concerns^(60)^ and one rated high risk of bias^(56)^, with the results summarised in Table 2. One study reported that daily maternal supplementation had no significant effect on vitamin D breast milk concentration^(56)^. The other four studies reported that a single large dose supplementation was more effective in raising breast milk vitamin D concentration than a smaller daily supplementation^(57–60)^. The supplements in the experimental studies varied from 50 µg per d to 3750 µg single dose.

#### Vitamin E

Six studies were included for vitamin E, with five experimental^(61–65)^ and one observational^(66)^, and are summarised in Table 2. Among the experimental studies, two were low risk^(61,63)^, one was rated with some concerns^(62)^ and two were high risk of bias^(64,65)^, whereas the observational study was considered good quality^(66)^.

Maternal intake of vitamin E (α-tocopherol) was shown to influence breast milk vitamin E concentration. The supplements in the experimental studies ranged from 40 mg/d to 536 mg in a single dose.

#### Vitamin K

Three experimental studies measuring vitamin K were included^(67–69)^, as summarised in Table 2. One was low risk of bias^(68)^, and two were considered high risk of bias^(67,69)^. The three studies reported that supplementing lactating mothers with vitamin K produced an increase in hind milk, foremilk and total breast milk vitamin K concentration, with a peak 12–24 h after supplementation. Vitamin K supplements varied from 0·5 to 5 mg per d for a period of 1 d up to 12 weeks.

### Water-Soluble vitamins

#### B vitamins

Five experimental studies were included^(70–74)^, one was rated as low risk of bias^(71)^, one presented some concerns^(72)^ and three were high risk of bias^(70,73,74)^, and are summarised in Table 2. Two studies investigated vitamins B_1_ and B_2_^(71,72)^, four investigated vitamin B_6_^(70,71,73,74)^ and one investigated vitamin B_12_^(74)^. The effects of maternal vitamin B_1_ intake on breast milk levels showed mixed results^(71,72)^. When the maternal supplementation was 1·7 mg/d for 6 weeks from parturition, there was no significant impact on vitamin B_1_ breast milk concentration^(72)^, whereas a supplement of 5 mg and then 10 mg over 2 d increased vitamin B_1_ content of breast milk.

Maternal vitamin B_2_ and B_6_ supplementation increased the breast milk vitamin B_2_ and B_6_ concentrations, respectively, in the first few postpartum weeks^(70–72)^. Maternal supplementations were 2 mg per d for vitamin B_2_ and ranged from 4 mg to 20 mg per d for vitamin B_6_ and lasted between 3 d and 6 weeks. Although, vitamin B_6_ maternal intake positively impacts breast milk concentration in the first few weeks postpartum, the effects of vitamin B_6_ supplementation were shown to decrease after 40 d^(74)^.

Vitamin B_12_ maternal supplementation did not show a significant effect on breast milk content at 1 week postpartum; however, a daily 8 μg intake was shown to prevent its decline in breast milk over lactation^(74)^.

#### Vitamin C

Three experimental studies were included on vitamin C^(74–76)^, all were rated high risk of bias, as summarised in Table 2. Overall, vitamin C in breast milk was only shown to be responsive to maternal intake following high-dose supplementation, that is, 1000 mg per d for 4 months^(76)^. Supplementation at lower doses, for example, 90 mg/d of vitamin C given to lactating mothers for 6 weeks, showed no difference in breast milk composition^(74,75)^

### Minerals (iodine, iron, copper, zinc and selenium)

#### Iodine

Four studies^(77–80)^were identified investigating iodine content, three were experimental^(77–79)^ and one observational^(80)^, as summarised in Table 3. Of the experimental studies, two were rated as low risk of bias^(78,79)^, and one high risk of bias^(77)^, whereas the observational study was rated of good quality^(80)^.

Maternal iodine supplementation increased breast milk iodine content and prevented a decline over lactation. Supplementation varied between 75 μg and 150 μg/d or a single 450 μg dose. The observational study found a positive correlation between breast milk iodine content and the consumption of at least three dairy products per d^(80)^.

#### Selenium

Six publications^(81–86)^ were included on Se, four RCT^(82–85)^ and two observation studies^(81,86)^, as summarised in Table 3. Among the experimental studies, one presented some concerns^(82)^, and three were rated high risk of bias^(83–85)^. The two observational studies were good quality^(86)^ and fair quality^(81)^. Maternal Se supplementation increased breast milk Se concentration. The experimental studies supplements varied from 20 μg/d, 50 μg/d and 200 μg/d for 3 months. One study^(82)^reported that maternal Se supplementation (50 μg per d) increased breast milk PUFA levels by 41 % (including LA and ARA) and decreased the levels of SFA by 11 %.

#### Iron, copper and zinc

One experimental study was included for Fe, which was rated low risk of bias^(87)^. Three observational studies were included, which measured Cu, Fe and Zn^(88–90)^. One was rated good quality^(88)^, and two were fair quality^(89,90)^, summarised in Table 3.

Cu, Fe and Zn maternal intake in healthy non-deficient lactating women was not shown to impact breast milk composition in the observational studies. Furthermore, in the experimental study, Fe supplementation at 80 mg/d for 4 months did not increase breast milk Fe levels^(87)^.

### Protein, ovalbumin, choline and tyrosine

#### Protein (amino acids)

Two publications were included^(91,92)^, one experimental study, which was rated as presenting concerns^(91)^, and one observational study^(92)^, which was rated as poor quality. Results are summarised in Table 3. There was no significant difference in the breast milk true protein, lacto-ferrin, α-lacto-albumin and lactose content between lactating women consuming a low or a high protein diet for 4 d^(91)^. However, breast milk from vegans was shown to have a lower taurine concentration than that from omnivores^(92)^

#### Ovalbumin

Two experimental studies^(93,94)^ were included, one was rated as some concerns^(94)^ and the other high risk^(93)^, summarised in Table 3. A direct dose response between the number of cooked eggs ingested and the ovalbumin concentration in breast milk was identified.

#### Choline

One observational study rated fair quality^(95)^ was included for choline, and the results are summarised in Table 3. The study reported differences in breast milk choline forms for vegans, as they had a greater mean concentration and distribution of choline derived from glycerophosphocholine than vegetarian and omnivores. Also, there was a lower mean percentage of choline from phosphocholine in vegan breast milk compared with vegetarian and omnivores.

#### Tyrosine

One experimental study, rated with some concerns, was included on tyrosine^(96)^. Results are summarised in Table 3. The study reported that lactating women supplemented with tyrosine had a higher breast milk total tyrosine concentration. The supplementation was a single dose of 10 g of tyrosine.

### Contaminants

Five observational studies were included^(89,97–100)^. One on Hg, which was rated good quality^(100)^, one on Hg and Pb, which was rated good quality^(99)^,one on As, B and Li, rated as fair quality^(97)^, one on Pb and Cd, rated as fair quality^(89)^, and one on PCB, rated as poor quality^(98)^. The results are summarised in Table 4.

### Heavy metals (arsenic, boron, cadmium, lead, lithium and mercury)

Maternal intake of freshwater fish was shown to be negatively associated with breast milk Hg levels, whereas maternal consumption of cereals was associated with higher breast milk Hg levels^(100)^.

One study identified a significant association between fish consumption and breast milk Pb levels^(99)^. The other studies show that environment can have a bigger impact on the presence of contaminants in breast milk than dietary habits^(89,97)^.

### Polychlorinated biphenyls

The reviewed study reported Inuit breast milk samples (300 g/d seafood intake) had a content in total 2,3,7-tetrachlorodibenzo-p-dioxinequivalents (TEQ) for PCB 3·5 times higher than Caucasian breast milk samples (12 g/d seafood intake)^(98)^.

### Overall summary

Table 5 provides an overall summary of the results of this systematic review and provides ratings of the overall quality of the evidence by the authors using the GRADE system for each nutrient and contaminant^(18)^. The table also summarises the doses of supplementation provided in the experimental studies, and where relevant European Food Safety Authority recommended intake levels are provided for comparison, as well as toxicity information on contaminant levels^(101)^.

**Table 5.** Synthesis of the nutrients in breast milk responsive to maternal diet

Nutrients	Number of articles	Relevance	Quality of evidence	Observations
PUFA/SFA				
DHA	29 (15 exp, 14 obs)	Essential for optimal brain and visual system development	High quality	DHA levels in BM proportional to maternal dietary DHA intake Maternal diet quality affects the fatty acid composition of breast milk. Lower DHA and long-chain fatty acids proportion in vegan BM For RCT, supplementation ranged from 200 mg to 1200 mg/d EFSA adequate DHA intake: 100–200 mg/d
EPA		Important for cardiovascular function and precursor of bioactive mediators with anti-inflammatory and pro-resolving properties	High quality	EPA levels in BM less proportional to maternal dietary EPA intake Effect of maternal EPA supplementation on BM concentration is more modest than DHA For RCT, supplementation ranged from 70 mg to 300 mg/d EFSA adequate EPA intake: 100–200 mg/d
ALA		Precursor of longer-chain *n*-3 PUFA and may have independent roles	Low quality	BM ALA content correlated with intake of rapeseed and soyabean oils; seeds and nuts such as flaxseed, chia and walnuts and some green leafy vegetables (kale and spinach) For RCT, supplementation was 10·1 g/d EFSA adequate intake: 0·5 % Energy intake of ALA
ARA		Essential for brain development and immune system function	Low quality	No effect observed in studies, but very low doses provided Same proportion of ARA in omnivores and vegans’ BM For RCT, supplementation was 54 mg/d EFSA adequate intake: Not available
SFA/TFA			High quality	Total trans acids (TFA) in the milk appeared to follow dietary trans changes after a 12–36 h lag period. No association between TFA consumption and BM TFA level Overweight women’s BM compared with normal-weight women’s BM contained higher amount of SFA, lower ratio of unsaturated to saturated FA than those of normal weight EFSA adequate intake: not applicable
Vitamins				
A	8 exp	Optimum visual, growth, immune system and cognitive development	High quality	Vitamin A level in BM is responsive to maternal intake Higher deficiency risks in developing countries For RCT, supplementation was either 30 mg/d, 54 mg/d or 60 mg single dose EFSA average requirement: 1020 µg RE/d
B_1_, B_2_ and B_9_	6 (5 exp, 1 obs)	B_1_ deficiency can cause gastrointestinal symptoms, cardiac failure, and lactic. B_2_ deficiency can cause anaemia and cataracts can develop if riboflavin deficiency is severe and prolonged B_5_ deficiency may include symptoms such as fatigue, insomnia, depression, irritability and vomiting B_9_ plays an essential role in the development of a baby’s brain and spinal cord during pregnancy.	Very low quality	Thiamine supplementation did not significantly affect the thiamine concentration in BM Riboflavin supplementations seem to have a positive impact on BM riboflavin concentration No evidence found on folic acid and pantothenic acid For RCT, vitamin B_1_ supplementation was 1·7 mg/d For RCTs, vitamin B_2_ supplementation was 2 mg/d EFSA average requirement vitamin B_1_:0·072 mg/MJ *Thiamin requirement is related to energy requirement and therefore expressed in mg/MJ. Values expressed in mg/d can be calculated based on the energy requirement of the group considered (EFSA NDA Panel, 2013)* EFSA adequate requirement vitamin B_2_:1·7 mg/d EFSA adequate intake vitamin B_5_:7 mg/d EFSA adequate requirement vitamin B_9_:380 µg DFE/d
B_6_		Essential for brain development	Moderate quality	Vitamin B_6_ supplementation seems to impact vitamin B_6_ breast milk concentration positively For RCT, Vitamin B_6_ supplementation ranged from 2·5 to 20 mg/d EFSA average requirement: 1·4 mg/d
B_12_		Essential for brain development	Moderate quality	Maternal vitamin B_12_ supplementation may prevent declines in BM content For RCT, Vitamin B_12_ supplementation was 8 µg/d EFSA adequate intake: 5 µg/d
C	3 exp	Essential for collagen formation, and role in immune system and nervous system function	Moderate quality	BM vitamin C levels responsive to maternal supplementation only when lactating mothers have low intake. Overall, vitamin C levels appear responsive to intake, but levels are regulated, as response to a highest dose of vitamin C was modest in European women in contrast with the 3-fold increase in African women. For RCT, Vitamin C supplementation ranged from 90 mg/d to 1000 mg/d EFSA average requirement: 140 mg/d
D	5 exp	Essential for Ca absorption and skeletal growth, deficiency can cause nutritional rickets	Moderate quality	BM vitamin D levels responsive to maternal supplementation. Higher effect when high unique dose supplementation (150 000 μg) For RCT, vitamin D supplementation ranged from 50 µg/d to 3750 µg single dose EFSA adequate intake: 15 µg/d
E	6 (5 exp, 1 obs)	Deficiency can compromise immune system and affect lung development. Premature infants are more susceptible to deficiency and can cause thrombocytosis, haemolytic anaemia and interventricular haemorrhage	High quality	Dose–response relationship, and supplementation effects are confirmed for α-tocopherol Milk total tocopherol found to be associated only with mother’s total fat and saturated fat dietary intake An increase in PUFA content may increase peroxidation and therefore increase vitamin E requirements. For RCT, Vitamin E supplementation ranged from 40 mg to 536 mg single dose EFSA adequate intake: 11 mg/d
K1	3 exp	Deficiency can lead to classical haemorrhagic disease of newborn with bleeding in the first week of life	Low quality	Dose–response relationship, and supplementation effects are confirmed For RCT, vitamin K supplementation ranged from 0·5 mg/d to 5 mg/d EFSA adequate intake: 70 µg/d
Minerals and others				
Iodine	4 (1 exp, 3 obs)	Deficiency can damage the developing brain and increase mortality	High quality	Dose–response relationship, and supplementation effects are confirmed Supplementation effects are immediate (peak 6 h after supplementation) For RCTs, iodine supplementation ranged from 75 µg/d to 150 µg/d. One study administrated 450 µg as a single dose EFSA adequate intake: 200 µg/d
Se	6 (4 exp, 2 obs)	Deficiency associated with increased respiratory morbidity	Low quality	BM Se significantly correlated to current fresh fish consumption, no significant correlation between intake of multivitamin supplements during pregnancy and Se in BM Se maternal supplementation seems to increase Se levels in BM and prevent decline with advancing lactation and also appeared to increase the BM concentration of PUFA (especially LA) and decrease BM SFA. For RCT, Se supplementation ranged from 20 µg/d to 200 µg/d EFSA adequate intake: 85 µg/d
Fe	4 (1 exp, 3 obs,)	Fe deficiency anaemia is associated with long-lasting neurofunctional effects and can affect sleep pattern	Low quality	Fe supplementation to non-anaemic women did not change BM Fe content or prevent a decline. For RCT, Fe supplementation was from 80 mg/d EFSA average requirement: 7 mg/d
Zn and Cu	3 obs	Zn deficiency can lead to cutaneous signs, diarrhoea and alopecia. Cu deficiency can cause bone lesions	Low quality	Dietary intake of Zn, Cu and Fe did not affect BM Zn, Cu and Fe concentrations. All metals with exception of Cu were found in lower concentrations in transitory milk. EFSA adequate intake Cu: 1·5 mg/d EFSA average requirement: Zn: 2·4 mg/d
Ovalbumin	2 exp	Exposure to ovalbumin may reduce egg allergy development	Moderate quality	Direct dose–response between the amount of cooked egg ingested and the BM ovalbumin For RCT, egg intake ranged from no egg to > 4 eggs/week EFSA adequate intake: Not available
Protein and amino acids	2 (1 exp, 1 obs)	Maternal protein consumption may impact the BM nitrogen content. BM nitrogen is used by beneficial bacteria present in the infant’s digestive system. Taurine has an important role in intestinal fat absorption, hepatic function, and auditory and visual development in preterm and low birth weight infant	Low quality	Output of lactoferrin, α-lactalbumin and serum albumin seem to be higher when a high protein diet is consumed; however, differences are not significant compared with low protein diet consumption. Mean taurine concentration in the vegan BM 35 mg/dl was significantly lower than in BM of omnivores. EFSA average requirement: Zn: 15 mg/d from 0 to 6 months PP and 10 g/d after 6 months PP
Tyrosine	1 exp	Tyrosine deficiency may increase risk postpartum depression	Low quality	Rise in free, but not total tyrosine, supplementation effects immediate (peak 6 h after supplementation) For RCT, tyrosine supplementation ranged from 2 to 10 g single dose EFSA adequate intake/average requirement: not available
Choline	1 obs	Needed for growth and development, and role in membrane and signalling functions	Low quality	No significant effects by maternal diet pattern. EFSA adequate intake: 520 mg/d
Contaminants				
As, Cd, Pb, Hg	4 obs	Heavy metals disrupt cellular events including growth can induce cancer	Moderate quality	Factors significantly related to metal levels in BM were area of residence (Hg, Pb), prematurity (Hg), fish consumption (Pb) and cereals (Hg), vitamin supplementation (Hg) and smoking (Pb, Cu). BM Pb positively associated with Fe and Zn but inversely associated with Ca. As in BM associated with Fe and B with Ca. No significant differences were found in BM between fish and non-fish eaters for THg concentrations. Negative association was found between THg concentrations in BM and freshwater fish consumption PTWI (JEFCA): no appropriate safety guidelines for As and Pb; Cd PTWI, 2·5 µg/kg bw/week; Inorganic Hg (IHg), 4 µg/kg bw/weeks. Total Hg (MeHg), 1·6 µg/kg bw/week
PCB	1 obs	PCB can induce skin and liver damage	Moderate quality	Levels were three times higher for very high seafood consumers (300 g/d in average) than for low seafood consumers (12 g/d in average) EFSA tolerable intake: 2 picograms per kg bw

Based on GRADE rating system.

Obs, observational study; exp, experimental study (randomised control trial); ALA, *α*-linolenic acid; ARA, arachidonic acid; BM, breast milk, FA, fatty acid; PP, postpartum; DFE, dietary folate equivalent; EFSA, European Food Safety Authority; THg, total Hg; PCB, polychlorinated biphenyls; PTWI, provisional tolerable monthly intake; bw, body weight; JEFCA, joint FAO/WHO Expert Committee on Food Additives; RE, retinol equivalent.

## Discussion

This study systematically reviewed the literature investigating the relationship between maternal intake and the levels of macronutrients, micronutrients and contaminants (heavy metals and PCB) in breast milk, for women without nutrient deficiencies. Due to the high heterogeneity between studies, it was not possible to undertake a meta-analysis, and so the results have been summarised with a narrative synthesis. The main findings were that there was strong evidence of response to maternal intakes of DHA, EPA, vitamins A, E and K, iodine and Se in breast milk composition, some evidence of response for ALA, B vitamins, vitamin C and D, ovalbumin, tyrosine and some contaminants, and insufficient evidence to determine the effects of ARA, Cu, Fe, Zn and choline. However, it should be noted that only a high dose of vitamin C was shown to produce an increase in breast milk vitamin C content, and although ARA intake was not found to affect breast milk ARA content, the supplemental dose used in this study was too low to allow definitive conclusions. Although maternal intake of Fe, Cu, Zn and total choline levels was not shown to affect their levels in breast milk, these findings are based on a limited number of studies, and so there remains uncertainty for these nutrients.

Maternal intake of DHA, EPA, ALA, B vitamins, and vitamins A, E, and K, iodine, Se, ovalbumin, and tyrosine was shown to affect their breast milk levels; however, the strength of evidence and quality of studies underpinning this evidence were highly variable. The relationship between DHA and EPA intake and breast milk levels has been extensively investigated, and the results across the experimental and observational studies show clear and consistent results that maternal intake influences their levels in breast milk^(19,20,22–25,29–33,35,36,38,40,43–47)^. With vitamin A, there was a much greater heterogeneity in experimental study design, particularly around the type of intervention and the dose and duration of supplementation; however, of the nine RCT, eight identified a positive relationship between intake and breast milk levels^(48–52,54,55)^. There have been fewer studies with B vitamins, and these are highly heterogeneous in study design, but overall, the results support the importance of maternal intake in influencing their levels in breast milk. Since the completion of this review, a relevant study has been published exploring the relationship between diet and nutritional status and the nutritional composition of donor milk^(102)^. Their results are consistent with our findings of a dose–response relationship between DHA intake and milk DHA content, and associations between maternal intake and milk levels of vitamins B_1_, B_2_, B_6_, C, and D. However, further high-quality studies are still needed, particularly for B_12_, to explore the effects of dose and also interactions between B vitamins^(70–74)^.

Studies of the effects of vitamin D intake are potentially confounded by seasonal effects, as breast milk concentrations of vitamin D and 25-hydroxyvitamin D have been shown to have significant seasonal variations^(103)^. Five experimental studies were identified that investigated the effects of maternal vitamin D supplementation on breast milk levels^(56–60)^. Overall, the strongest effects were identified with the higher doses of supplementation, and importantly supplementation was shown to alleviate seasonal declines. It may therefore be advisable to recommend vitamin D supplementation in circumstances where endogenous synthesis is limited.

In all five experimental studies, maternal vitamin E supplementation increased breast milk *α*-tocopherol levels; however, two studies directly compared natural (RRR *α*-tocopherol) and synthetic (all-racemic *α*-tocopherol) sources^(61,63)^. Synthetic *α*-tocopherol is an equimolar mix of its eight stereoisomers, as the three chiral carbons of *α*-tocopherol can be in either an R or an S orientation, whereas in nature only one of these isomers (RRR) is found^(104)^. Clemente and co-workers found that supplementation with both forms increased vitamin E concentrations in breast milk (colostrum); however, the RRR form was more efficient in increasing the levels^(61)^. Gaur and co-workers reported that supplementation with RRR increased the percentage of RRR *α*-tocopherol isomers in breast milk, whereas supplementation with all-racemic *α*-tocopherol decreased the percentage of RRR stereoisomers and increased the non-RRR-*α*-tocopherol stereoisomers, such as 2S-*α*-tocopherol^(63)^. Since the relative effects and potencies of these different forms of *α*-tocopherol on health outcomes are not well understood^(104)^, it may be prudent at this time to recommend the RRR form of *α*-tocopherol, where supplementation is advised.

Three experimental studies were identified for vitamin K, and although a wide range of supplementation protocols were employed, a consistent dose–response relationship was identified between maternal vitamin K intake and breast milk levels^(67–69)^. Three experimental and one observational studies were identified that explored the relationship between maternal intake of iodine and breast milk levels, and a consistent relationship was identified across all studies^(77–80)^. With Se four experimental studies were identified, and they all showed that maternal supplementation increases breast milk levels^(82–84)^; however, the observational studies were discordant, with one identifying a significant association between fish intake and breast milk Se levels^(86)^, whereas the other did not identify any significant associations^(81)^.

No clear relationship between nutrient intake and breast milk levels was identified for Cu, Fe and Zn; however, this is based on a limited number of studies^(87–90)^. The only experimental study reported that Fe supplementation of non-anaemic women did not increase breast milk Fe levels^(87)^, whereas although one observational study found that breast milk Fe concentration was significantly higher from those reporting taking daily Fe supplements^(88)^, the other did not identify an association between Cu, Fe and Zn intake and breast milk levels^(89)^. Albeit with this paucity of evidence, these results are consistent with previous observations that breast milk Cu, Fe and Zn concentrations are not associated with maternal mineral status^(105)^.

Overall, the observations from the present review are consistent with the results of previous systematic reviews in this area^(6–9)^, with the exception of vitamin C, where we did not identify a clear relationship between maternal intake and breast milk levels. This dissonance between may be based on differences in the study selection criteria between systematic reviews, as the present review only included studies on non-deficient populations. In the present review, three experimental studies were identified for vitamin C^(74–76)^. All three studies were based on small numbers of participants, with a range of different supplemental dosing regimens administered. A relationship between maternal intake and breast milk content was only identified in one trail, which provided vitamin C at high doses, that is, 1000 mg per d^(76)^. It may therefore be hypothesised that in non-deficient populations, breast milk vitamin C levels are only responsive to higher levels of maternal supplementation; however, this needs further investigation.

It is important to highlight that in most of the reviewed experimental publications, the nutrient doses were higher than the EFSA adequate intake or average requirements^(101)^. For instance, in all nine reviewed experimental studies, DHA and EPA supplementation was shown to increase breast milk DHA and EPA levels, and this was in a dose-dependent manner^(19,20,22–25,29,31,33)^. The highest dose of DHA provided was 1·2 g for 14 d. The levels of supplementation provided were also higher than EFSA recommendations for vitamins A, B_6_, B_12_, E and K^(101)^.

With regard to specific restrictive diets, breast milk from vegan mothers contained low DHA levels^(44)^, and in one study this was significantly lower than breast milk from vegetarian and omnivore mothers^(46)^. The choline composition profile was also reported to be lower in breast milk from vegan mothers^(95)^, and taurine was also found to be lower in breast milk from vegan mothers^(92)^. Although the present review did not identify any studies that compared vitamin B_12_ content between mothers following vegan, vegetarian and non-vegetarian diets, vitamin B_12_ content was shown responsive to maternal intake. Therefore, our results support the previous recommendations in the systematic review by Karzc and Krόlak-Olejnik, that mothers following a vegan diet should consider supplementation with preformed DHA and vitamin B_12_, as maternal levels of intake of these nutrients may be low^(8)^. Furthermore, as vegan and vegetarian diets become more popular, there is an urgent need to conduct further high-quality studies in this area, so lactating mothers and milk bank donors can be provided with specific nutritional recommendations.

With regard to the effects of intake of contaminants such as heavy metals and PCB, and breast milk levels, all studies reviewed were observational in nature, and it should be noted that any observed effects may therefore be confounded by environmental factors in addition to dietary intake^(89,97)^. However, two studies reported a negative relationship between freshwater fish consumption and breast milk Hg content, and a positive relationship was found with cereals and vitamin supplements consumption^(99,100)^. A positive relationship was reported between maternal fish consumption (especially large fish species) and PCB content in breast milk, although the fish intake was extremely high (300 g/d)^(98)^. Overall, the number of studies published in this area is limited, with very few publications focusing on dietary sources; however, the overall presence of contaminants in breast milk appears below toxicity levels. Furthermore, based on these observations, fish intake was not identified as a potential source of elevated Hg in breast milk.

A strength of this systematic review is the extensive scope of nutrients and contaminants that have been considered, and recommendations in some areas are possible. Furthermore, our results have identified areas where there is a lack of high-quality evidence, particularly around the effects of ARA, Cu, Fe and Zn supplementation on breast milk content. The relationship between contaminant intake and breast milk levels also requires more comprehensive analysis, to delineate the effects of dietary intake from wider environmental exposures. A further strength is that this review considered studies undertaken in populations from a wide variety of countries and ethnicities, where healthy non-micronutrient-deficient lactating women were considered. Publications from developing countries were not included, unless it was clearly specified that the participants did not have a nutrient deficiency. However, as many publications on B vitamins were excluded during the selection process, as they were reporting on either deficient or low-income populations, we could not include any studies on vitamin B_5_ or folic acid.

Breast milk is a highly dynamic fluid, and the nutritional content has been shown to be affected by physiological factors in addition to nutritional intake, such as stage of lactation^(106)^ and circadian rhythm^(107)^. Due to the variability in study reporting and high degree of design heterogeneity, it was not possible to draw specific conclusions about the effects of these factors in our analysis. Furthermore, other uncontrolled covariates that have been shown to influence the target compounds in breast milk and that were not analysed in the review, such as environment, obesity or genotype. For example, biosynthesis of DHA is influenced by genotype, as variations in SNP, particularly the fatty acid desaturase (FADS) gene cluster, affect PUFA levels. SNP within this cluster impact on expression of FAD genes across a wide range of tissues are associated with variations of *n*-6 and *n*-3 PUFA levels, such that those with the minor FADS allele have higher levels of LA and lower levels of ARA and DHA in serum phospholipids, plasma phospholipids and breast milk compared with carriers of the major allele^(108–110)^. Importantly, it has been shown that only women with the major allele appear to increase breast milk DHA by consuming fish or fish oil^(110)^.

It should also be highlighted that the observational studies employed a wide variety of tools to assess dietary intake, and these have varying levels of precision, and there was also very little information across the publications as to how the supplements were ingested, and therefore absorbed, as it has been demonstrated that lipid-soluble nutrients are better absorbed when ingested with a fat meal^(111)^. These aspects should be considered in the design of future studies and also when providing nutritional advice to lactating mothers and breast milk donors. Our review limited the contaminants list to heavy metals and PCB; however, other ingredients should be further investigated. This is the case of non-nutritive sweeteners that were recently found in breast milk from lactating women in the USA^(112)^.

In conclusion, the present systematic review assessed the available literature on quantitative associations between maternal diet and breast milk composition. Maternal intake, particularly DHA, EPA, ALA, and vitamins A, D, E, B_6_, B_12_ and K, ovalbumin and tyrosine were found to be responsive to maternal diet, whereas there is insufficient evidence to ascertain the effects of intake on breast milk ARA, vitamin C, Fe, Zn, Cu and choline. Although these results provide information that can be used to help inform nutritional guidelines for lactating mothers and breast milk donors, further high-quality research is needed to inform nutrient intake recommendations. At present, the recommendation for lactating women and milk donors should adopt a healthy and diversified diet, such as the Mediterranean diet, and consider supplementation with nutrients such as DHA and vitamins B_12_ and D when their diets are restricted or limited by external factors.

## Supporting information

Falize et al. supplementary material Falize et al. supplementary material

## Supplementary material

For supplementary material/s referred to in this article, please visit https://doi.org/10.1017/S0007114523002775
