# Supplementary material for: Evaluating the relationship between the nutrient intake of lactating women and their breast milk nutritional profile: a systematic review and narrative synthesis
Source: Br J Nutr. 2023 Dec 6;131(7):1196–224. doi: 10.1017/S0007114523002775 (PMC10918524; doi:10.1017/S0007114523002775)
Supplement: Falize et al. supplementary material [file S0007114523002775sup001.docx]

Supplementary Table 1: Search strategy

| Search Number | Query |
| --- | --- |
| 1 | Breastfeeding |
| 2 | Lactating women OR Lactation |
| 3 | Human milk nutritional profile OR human milk composition OR Maternal nutritional factors |
| 4 | Human milk donor |
| 5 | Breast milk OR Human milk OR Mother Milk OR Maternal milk |
| 6 | Maternal nutrition OR Maternal Diet |
| 7 | #1 OR #2 OR #3 OR #4 OR #5 OR #6 |
| 8 | Vegan Diet |
| 9 | Vegetarian Diet |
| 10 | Low carb Diet OR Ketogenic Diet |
| 11 | Micronutrient |
| 12 | Macronutrient |
| 13 | Supplement |
| 14 | Niacin OR nicotinamide OR Vitamin B3 |
| 15 | Pyridoxine OR Vitamin B6 |
| 16 | Cobalamin OR Vitamin B12 |
| 17 | Thiamine OR Vitamin B1 |
| 18 | Riboflavin OR Vitamin B2 |
| 19 | Pantothenic acid or Vitamin B5 |
| 19 | Retinol OR beta carotene OR Vitamin A |
| 20 | Folate OR folic acid OR folacin, OR Vitamin B9 |
| 21 | Ascorbic Acid OR ascorbate OR Vitamin C |
| 22 | Cholecalciferol OR Ergocalciferol OR 25-hydroxycholecalciferol OR 25-hydroxyvitamin D OR Vitamin D |
| 23 | Tocopherol OR Vitamin E |
| 24 | Iodine |
| 25 | Iron OR lactoferrin |
| 26 | Protein OR Amino Acids |
| 27 | Zinc |
| 28 | Copper |
| 29 | Selenium |
| 30 | Tyrosine |
| 31 | Choline |
| 29 | Docosahexaenoic acid OR DHA OR DPAn-3 OR DPAn-6 |
| 30 | Arachidonic Acid OR ARA |
| 31 | Eicosapentaenoic Acid OR EPA |
| 32 | Alpha Linolenic Acid OR ALA |
| 33 | Linoleic Acid OR LA |
| 34 | Adrenic acid OR AdA |
| 35 | Fish intake OR Marine oils |
| 36 | Long chain Polyunsaturated fatty acid |
| 37 | Polyunsaturated fatty acid OR PUFA OR LC PUFAs |
| 38 | Omega 3 OR n-3 OR n3 OR n3 PUFA |
| 39 | Omega 6 OR n-6 OR n6 OR n6 PUFA |
| 40 | Trans fatty Acids OR saturated fats OR Hydrogenated Fats |
| 41 | Toxic heavy metals OR Mercury OR Hg OR Aluminium OR Al OR Chromium OR Cd OR Arsenic OR As OR Lead OR Pb |
| 42 | #8 OR #9 OR #10 OR #11 OR #12 OR #13 OR #14 OR #15 OR #16 OR #17 OR #18 OR #19 OR #20 OR #21 OR #22 OR #23 OR #24 OR #25 OR #26 OR #27 OR #28 OR #29 OR #30 OR #31 OR #32 OR #33 OR #34 OR #35 OR #36 OR #37 OR #38 OR #39 OR #40 OR #41 |
| 39 | Randomised controlled trials OR randomized controlled trials |
| 40 | Observational studies |
| 41 | Experimental studies |
| 42 | #39 OR #40 OR #41 |
| 43 | #7 AND #42 AND #43 |

Supplementary Table 2: Breakdown per nutrient

|  | Experimental Studies | Observational Studies | Total |
| --- | --- | --- | --- |
| Fatty Acids | 15 | 14 | 29 |
| Vitamins | 29 | 2 | 31 |
| Minerals | 6 | 11 | 17 |
| Contaminants | 0 | 5 | 5 |
| Others | 4 | 2 | 6 |
